# Associations of brain structure with psychopathy

**DOI:** 10.1007/s00406-025-02028-6

**Published:** 2025-05-29

**Authors:** Peter Pieperhoff, Lena Hofhansel, Frank Schneider, Jürgen Müller, Katrin Amunts, Sabrina Weber-Papen, Carmen Weidler, Benjamin Clemens, Adrian Raine, Ute Habel

**Affiliations:** 1 Institute of Neuroscience and Medicine (INM-1), Research Centre Jülich, Jülich, Germany; 2 Department of Psychiatry, Psychotherapy and Psychosomatics, Medical Faculty, RWTH Aachen University, Aachen, Germany; 3 Institute of Neuroscience and Medicine (INM-10), Research Centre Jülich, Jülich, Germany; 4 Department of History, Philosophy and Ethics of Medicine, School of Medicine, Heinrich-Heine-University Düsseldorf, Düsseldorf, Germany; 5 University Hospital Düsseldorf, Heinrich Heine University Düsseldorf, Düsseldorf, Germany; 6 Department of Psychiatry, Forensic Psychiatry, Human Medical Center, Georg August University, Göttingen, Germany; 7 Departments of Criminology, Psychiatry, and Psychology, University of Pennsylvania, Philadelphia, PA USA; 8 C. and O. Vogt Institute for Brain Research, University Hospital Düsseldorf, Heinrich Heine University Düsseldorf, Düsseldorf, Germany

**Keywords:** Psychopathy, MRI, DBM, Morphometry, Frontal-subcortical circuits, Brain atlas

## Abstract

Psychopathy is one of the greatest risk factors for serious and persistent violence. In order to detect its neurobiological substrates, we examined 39 male psychopathic subjects and matched controls using structural MR imaging and the Psychopathy Check-List (PCL-R). Individual brain region volumes were calculated using the Julich-Brain and AAL3 atlases. Associations of region volumes with the PCL-R dimensions among psychopathic subjects and differences between both groups were analysed. PCL-R factor 2 assessing lifestyle and antisocial behaviour showed in the psychopathic sample negative associations with volumes of several regions, including pons, nuclei of basal ganglia, thalamus, basal forebrain (CH-4), cerebellar regions and areas in orbitofrontal, dorsolateral-frontal and insular cortices. These findings suggest dysfunctions in specific frontal-subcortical circuits, which are known to be relevant for behavioral control. In contrast, the interpersonal-affective PCL-R factor 1 showed only weak positive and negative associations with orbitofrontal, dorsolateral-frontal and left hippocampal areas (CA1, subiculum), among others, indicating that involved brain regions might be affected to a variable degree in different individuals. The group comparison yielded a significantly reduced total brain volume in psychopathic subjects relative to controls, while pronounced regional focuses of volume differences were found only in the right subiculum, suggesting an interindividually variable pattern of structural deviations in the brains of psychopathic subjects. In conclusion, these findings are compatible with the dimensionality of the PCL-R construct, and suggest a particulary strong association of antisocial behavior to smaller volumes in widespread subcortical-cortical brain regions.

**Supplementary Information:**

The online version contains supplementary material available at 10.1007/s00406-025-02028-6.

## Introduction

Psychopathy is a personality construct that represents one of the strongest predictors of aggressive and violent behavior [1]. Individuals with strong psychopathic traits commit more crimes, cause greater injuries and have a higher risk of recidivism compared to non-psychopathic subjects [2, 3], posing a high financial burden to society and psychological and physical burden to victims. Psychopathy is conceptualized by affective and interpersonal dysfunctions and antisocial behavior [4] that is not explicitely categorized by ICD 10 or DSM 5 as personality disorder and is not completely identical with the diagnosis of antisocial personality disorder. The most commonly used instrument to assess psychopathy in clinical and forensic settings is Hare‘s Psychopathy Checklist (PCL-R) [5, 6], which yields a univariate total score, but also distinguishs two dimensions: The first factor of the PCL-R outlines interpersonal and affective problems, and is often referred to as the core feature of psychopathy. It becomes manifest in e.g., pathological lying, a grandiose sense of self-worth, and lack of empathy, remorse or guilt. Individuals in whom this factor is strongly expressed are often superficially charming, highly manipulative, show deficient emotional reactivity and shallow affect, high levels of predatory behavior and instrumental aggression [7]. Unable to experience deeper feelings, these individuals show a generally low emotional reactivity and, consequently, low levels of anxiety [8, 9]. The second dimension of the PCL-R describes impulsive and antisocial behavior, and is reflected in sensation seeking, irresponsible behavior, anger, parasitic and unstable lifestyles, as well as an early onset of deviant behavior and high recidivism [9–11]. Although both dimensions are closely related in the phenomenon of psychopathy, they comprise fundamentally divergent patterns of conduct and traits. Hence it is reasonable to assume that they are based on distinct neurobiological underpinnings.

Structural magnetic resonance imaging (MRI) has been used in several studies in order to find interindividual differences in brain structure which are associated with psychopathy. Structural measures were analyzed by group comparisons e.g. between psychopathic and non-psychopathic subjects, or regression with the PCL-R score. The findings of early studies, however, were only to a minor degree consistent, as was pointed out in two review articles [12, 13], presumably because of low sample sizes and heterogenous characteristics in particular of the psychopathic subjects. More recent overviews of imaging studies [4, 14–17] show that structural differences were mostly located in the grey matter, but regional localizations of different studies are still diverse and spread nearly over the whole brain.

Several studies reported predominantly negative associations between the PCL-R total score and the volume of the whole prefrontal cortex (PFC) [18], regions in orbitofrontal cortex (OFC) [19, 20] and amygdala [19]. Studies of associations between the two PCL-R main dimensions and brain structure suggest that the first factor, interpersonal and affective problems, may be primarily associated with gray matter volume (GMV) reductions in prefrontal, orbitofrontal and temporal regions [20–26], and to GMV reductions in medial temporal lobe structures such as the hippocampus [27] and the amygdala [21]. Similar reductions have also been found in a study comparing competitive judo athletes with non-athletes [28], which demonstrated the dimensionality of psychopathic traits without necessarily involving criminal or antisocial behavior. A meta-analysis [29] summarized the results of seven Voxel-based morphometry (VBM) [30] studies that had calculated regression analyses of whole brain morphology and psychopathy scores [21, 22, 26, 31–34]. The authors found throughout negative correlations of PCL-R factor 1 with GMV in the frontal cortex (bilateral OFC, left middle frontal gyrus, i.e., dorsolateral parts), somatosensory regions, motor regions (left SMA and precentral gyrus), left cingulate (middle and posterior parts) and the bilateral inferior temporal gyri.

Reported associations of the second PCL-R dimension, impulsive-antisocial behavior, are less consistent. Greater expressions of impulsive-antisocial behavior have been found to be positively correlated with volumes of the prefrontal and in particular orbitofrontal cortex [21, 23, 24, 35], as well as the insula, putamen and pallidum [24], whereas negative correlations with GMV have been observed in the temporal lobe, fusiform gyrus, insula, parahippocampal and occipital areas [20, 35, 36], as well as cingulate gyrus [36]. Other studies have failed to find significant correlations between impulsive-antisocial behavior and regional GMV [22, 26, 27].

The aforementioned VBM meta-analysis [29] found consistently negative correlations of the PCL-R factor 2 with GMV, located in the left middle frontal and orbitofrontal gyri, middle and posterior cingulate cortices, pre- and postcentral gyri and middle occipital regions, and bilaterally in the striatum and inferior and middle temporal cortices. Yet the results of this work highlight two major challenges of psychopathy research: First, the number of available studies is relatively low (only seven studies could be included), and second, the individual results of these studies are still quite incongruent. A major reason for this is that conducting neuroimaging studies with psychopathic subjects and especially with incarcerated criminal subjects is challenging. Hence, studies in this field often suffer from small sample sizes and low statistical power [29]. To circumvent this problem, researchers have often employed community samples with sub-clinical manifestations of psychopathy (e.g., university students), taking the risk that the results could be distorted by floor effects. Using different assessment tools and cut-off criteria for psychopathy can also influence the results [15]. Moreover, researchers have to contend with comorbidities that often occur in forensic populations, such as substance use disorders [33], borderline personality disorders [34] or schizophrenia [37].

For this study we selected individuals with a psychopathy score ≥ 20 assessed by the PCL-R from three German forensic cohorts [26, 38, 39] as well as age-matched control groups, in order to identify brain regions that can be associated with psychopathy. Structural MR images were analysed by deformation-based morphometry (DBM) to compute individual volumes of neuroanatomical regions which were defined by the Julich-Brain atlas of cytoarchitectonic regions [40]. Associations of these volume data with the PCL-R dimensions among psychopathic subjects and differences between both subject groups were computed. Our main hypothesis proposed that the behavioral traits forming the two PCL-R dimensions involve different neuronal circuits. Given that PCL-R factor 1 (interpersonal and affective problems) is associated with low emotional reactivity, low levels of anxiety and poor threat response, but could also require high cognitive functioning [41], we expected to find regions involved in emotion processing, such as limbic regions, to be negatively correlated with this factor, while areas responsible for cognitive control, particularly in the prefrontal and temporal regions, to be unaffected or even positively associated. PCL-R factor 2 (impulsive and antisocial behavior) on the other hand is defined by disturbed impulse control, substance misuse and an antisocial lifestyle and was hypothesized to correlate negatively with GMV primarily in the regulatory brain regions (e.g. in the prefrontal, orbitofrontal or temporal lobes).

## Methods

### Participants

Thirty-nine high scoring male psychopathic subjects with a total PCL-R score ≥ 20 and controls were selected from three German cohorts (Table 1): (1) MU [26] was recorded at the University Hospital in Göttingen, Germany. (2) FS, UH [39] was recorded at the Forschungszentrum Jülich, Germany. (3) UH, LH [38] was acquired in the Medical Faculty of RWTH Aachen University, Germany. Psychopathic and control subjects were matched by age within each dataset. Psychopathic subjects were recruited from forensic hospitals and local parole offices, whereas the respective control group was invited by public advertisement. All participants were German-speaking, right-handed males between 18 and 60 years of age, fulfilled the requirements to participate in an MRI study (e.g. no metal implants, no epilepsy) and had no history of psychiatric or neurological diseases, other than substance use disorders, as assessed by the German version of the Structured Clinical Interview (SCID I) for DSM-IV Disorders [42]. By their own admission, all participants were drug-free for at least six months prior to the assessment [26] or reported not having consumed any opiates for at least one year [38].

**Table 1:** Subsample characteristics and univariate group comparisons of psychopathic subjects (PS) and controls (C)

	UH, LH		FS, UH		MU		Statistics	
	C	PS	C	PS	C	PS	F (df)	*p*
N	12	12	10	10	17	17		
age in years	37.42 (11.70)	37.00 (11.00)	28.90 (7.98)	30.00 (7.53)	30.47 (7.63)	35.06 (9.11)	0.989 (1, 76)	0.323
PCL-R								
total		23.02 (2.14)		27.70 (4.62)		33.29 (4.44)	19.264 (2, 36)	< 0.001***
factor 1		10.22 (2.45)		11.6 (2.07)		13.71 (2.52)	7.763 (2, 36)	< 0.01**
factor 2		11.18 (2.55)		13.00 (1.94)		15.00 (2.09)	10.69 (2, 36)	< 0.001***
ICV in cm^3^	1641 (145)	1608 (131)	1605 (92)	1498 (56)	1648 (143)	1582 (99)	HC: 0.36 (2,36) PS: 3.47 (2,36)	0.701 < 0.05*

Characteristics of psychopathic subjects (PS) and controls (C), and univariate testing of differences in subsamples, reporting mean and standard deviation of each parameter, degrees of freedom (*df*) and *p*-value (*p*) of tests for differences in age between groups, differences in PCL-R scores between studies, and differences in ICV between studies, separately for each group. (significance coefficient: *p <.05; **p <.01; ***p <.001)

Written informed consent was obtained from each participant. The study protocols were in accordance with the Declaration of Helsinki and approved by the respective local Ethics Committees of the University of Regensburg, the Institutional Review Board of the Medical Faculty, RWTH Aachen University and by the Institutional Review Board of the University of Düsseldorf.

### Psychopathy assessment

Psychopathy was diagnosed using the revised German version of the PCL-R [43], which is a rating scale based on a semi-structured interview. The interview was conducted by a trained medical doctor or psychologist who rated the extent to which each of 20 items applied to the examined subject on an ordinal three-point scale (0 = does not apply; 2 = definitely applies) [44]. The PCL-R total score ranges from 0 to 40, reflecting the participants’ global psychopathic trait [45]. A cutoff value of 30 of the PCL-R total score had been suggested for the diagnosis of psychopathy in a North American prison population [44]. However, a meta-analysis of European offender samples found lower mean PCL-R scores than in their North American counterparts [43], suggesting the necessity to adjust the cutoff value. The applicability test of the PCL-R in a forensic sample in Germany revealed a mean total score of 19.33 [46]. Only 26% of the participants of that German sample met the criterion of a score equal to or greater than the sum score of 25, which was recommended for an international population [45, 47]. Based on these findings, we decided to include participants of the three data sets with a total PCL-R score of 20 or higher.

### Image analysis

Details about the applied MR acquisition techniques are reported in Supplementary material. The T1-weighted MR image data were analyzed by Deformation-Based Morphometry (DBM) which estimates the volumes of anatomically defined structures in each brain. The complete processing chain had been previously described in [48]. In short, brains were extracted from MR images using the automatical segmentation procedure of SPM12 [49]. Each extraction was visually inspected and corrected, if necessary, using the program ITK-SNAP [50] (www.itksnap.org*).* The extracted brain images were affinely registered with the single subject MNI Colin27 reference brain [51] using the program Flirt, which is part of the FSL package [52] (https://fsl.fmrib.ox.ac.uk/fsl/fslwiki/*)*, followed by a symmetrical non-linear registration. The latter yielded a deformation field for each brain, i.e. a vector field, from which a map of voxel-wise volume measures, defined by “local volume ratios” (LVR) [48] relative to the reference brain was derived. The JURECA supercomputer [53] in the Research Centre Jülich was used for the image registrations.

Next, volumes of anatomical regions of interest (ROI) were calculated in each individual brain by summation of the LVR values over the corresponding ROI in the reference brain (weighted by the local probabilistic value in case of probabilistic maps). The analysis of region volumes has two major advantages in comparison with a voxel-wise analysis: First, region-based volume estimates are more robust than voxel-wise estimates given the inevitable inaccuracies of voxel-level registrations. Second, anatomical knowledge about the regional structure of the brain is directly incorporated into the analysis and interpretation of findings.

For the regional analysis we applied a hierarchical atlas system to capture psychopathy related differences on multiple anatomical levels. This atlas system is based mainly on the Julich-Brain-Atlas version 3.1 [40] (https://www.ebrains.eu/tools/human-brain-atlas), which encompasses 454 cytoarchitectonic probabilistic maps of cortical areas and nuclei. They were complemented by maps of striatum and cerebellar lobules from the AAL3 atlas [54, 55], and masks of the mesencephalon, pons and cerebellar white matter. These *elementary* regions where combined to form maps of superordinate regions in order to model the anatomical hierarchy of the brain (e.g. cortical area-6d1 ⊂ dorsal precentral gyrus ⊂ frontal lobe ⊂ cerebral cortex ⊂ telencephalon ⊂ brain). Supplementary Table S6 shows the complete hierarchy of brain regions. The brain region hierarchy is used for the multiple-testing procedure to control the type-I error in the statistical analysis (see below).

The intracranial volume (ICV) of each subject was measured by manual segmentation as described in the Supplementary material.

### Statistical analysis

The brain morphology was analyzed using Analysis of Covariance (AnCoVa) models, with the region volume as the dependent variable. Because data of three different MR sites were assembled in this study, a categorical factor *study site* was part of each model. Group differences in region volumes between psychopathic subjects and controls were modelled with the predictor variables *group (psychopathic*,* control)*,* study site*,* age* and *ICV*. Associations between each region volume and the PCL-R factor 1 and 2 were analyzed by *one* AnCoVa model encompassing both factors as predictor variables (in accordance with [29, 56]) besides the variables *study site*,* age*,* ICV* within the group of psychopathic subjects. Thus the contribution of each PCL-R factor to the region volume could be tested after adjustment for the other factor in order to enhance the specificity of the associations. Associations with the PCL-R total score were separately analyzed. These analyses were carried out with the software SAS 9.4 (SAS Institute, Cary, NC).

In order to control the type-I error probability, we applied a recently developed statistical methodology [57–59], which aims to improve the statistical power for the testing of large numbers of hypotheses occuring e.g. in imaging or genomic studies. It is based on the partitioning of the *elementary* hypotheses into families, so that these families (each as a whole) are tested first, and subsequently the hypotheses within each family that has a significant effect. The arrangement of all hypotheses (i.e. tested brain regions) in a multi-level hierarchical tree (similar to [58]) matches the hierarchial structure of the brain, with the complete brain at the top level, and single areas or nuclei at the lowest level (see Supplementary Table S6), in order to capture dependencies among brain regions.

A region family is formed by those child regions which have the same “parent” in the tree. The p-value of a family of *N* hypotheses can be calculated by different methods (Supplementary material of [58]). A frequently used method is the Simes procedure [60]:

1 $$\:{p}_{Simes}=\underset{j=1,\dots\:,N}{{min}}{p}_{\left(j\right)}\cdot\:N\:/\:j$$

Here $\:{p}_{\left(1\right)}\le\:\:{p}_{\left(2\right)}\le\:\dots\:\:\le\:\:{p}_{\left(N\right)}$ are the p-values of the hypotheses *within* the family arranged in increasing order.

In case of spatial data an alternative method is to average or sum the spatial signal over a given family (i.e. region) and to assess the statistical test for this regional signal [61]. Since this study examined volumes of regions it is straightforward to calculate also the volumes of region families and test them directly with the statistical models described before. This method is more sensitive for diffuse, wide-spread effects, whereas the Simes procedure might be more sensitive for strong focal effects.

After having calculated the p-values of all regions (on all tree levels), the Benjamini-Hochberg procedure to control the False-discovery-rate (FDR) [62] is applied within each family (instead of among *all* regions in one step), beginning at the top-most level of the region hierarchy. Only those child regions are considered whose parent region had shown a “discovery” [58] (i.e. whose corresponding null hypothesis had been rejected). Moreover, the FDR threshold is reduced from level to level by the proportion of “discoveries” among all regions in the parent family:

2 $$\:{q}_{l}=\:{q}_{l-1}\cdot\:\#\mathrm{d}\mathrm{i}\mathrm{s}\mathrm{c}\mathrm{o}\mathrm{v}\mathrm{e}\mathrm{r}\mathrm{i}\mathrm{e}\mathrm{s}\:/\:\#\mathrm{c}\mathrm{h}\mathrm{i}\mathrm{l}\mathrm{d}\mathrm{r}\mathrm{e}\mathrm{n}$$

($\:{q}_{l},\:{q}_{l-1}$ = specified FDR thresholds at level *l* and *l-1*) [58]. The initial FDR threshold 0.05 was used for all analyses. For the group comparison the p-values of region families were directly calculated (method 2), whereas for the regression analyses with the PCL-R scores the Simes procedure was employed.

We note that the successive reduction of the FDR threshold is a rather strict requirement [63]. A somewhat relaxed criterion is to keep the FDR threshold constant at 0.05 on all levels but still requiring that a continous path of regions showing a signifcant “discovery” from the top level to each considered region exists.

## Results

### Group characteristics

Ages of psychopathic subjects (34.36 ± 9.53) and controls (32.21 ± 9.60) weren’t significantly different (*p* =.323, *Cohen’s d* = 0.224). The mean PCL-R scores of psychopathic subjects were PCL-R total = 28.70 ± 5.88, factor 1 = 12.09 ± 2.79 and factor 2 = 13.31 ± 2.71. The PCL-R scores of control subjects had not been assessed. Sample characteristics are presented in Table 1. The correlation of PCL-R factor 1 and 2 was 0.27 with *p* =.0963, i.e. non-significant. This is less than the correlations reported in other studies (e.g. *r* =.51 in [20] with PCL-R total = 3.2–37.6, or *r* =.39 to 0.54 in [64]), however these estimates were based on much larger samples, and the PCL-R factor values variied over larger ranges than in our sample.

### Structural analyses

For the neuroanatomical terms of areas and nuclei we refer to Supplementary Table S6.

#### Associations of region volumes with PCL-R factor 1 (interpersonal and affective problems)

**Fig. 1:**
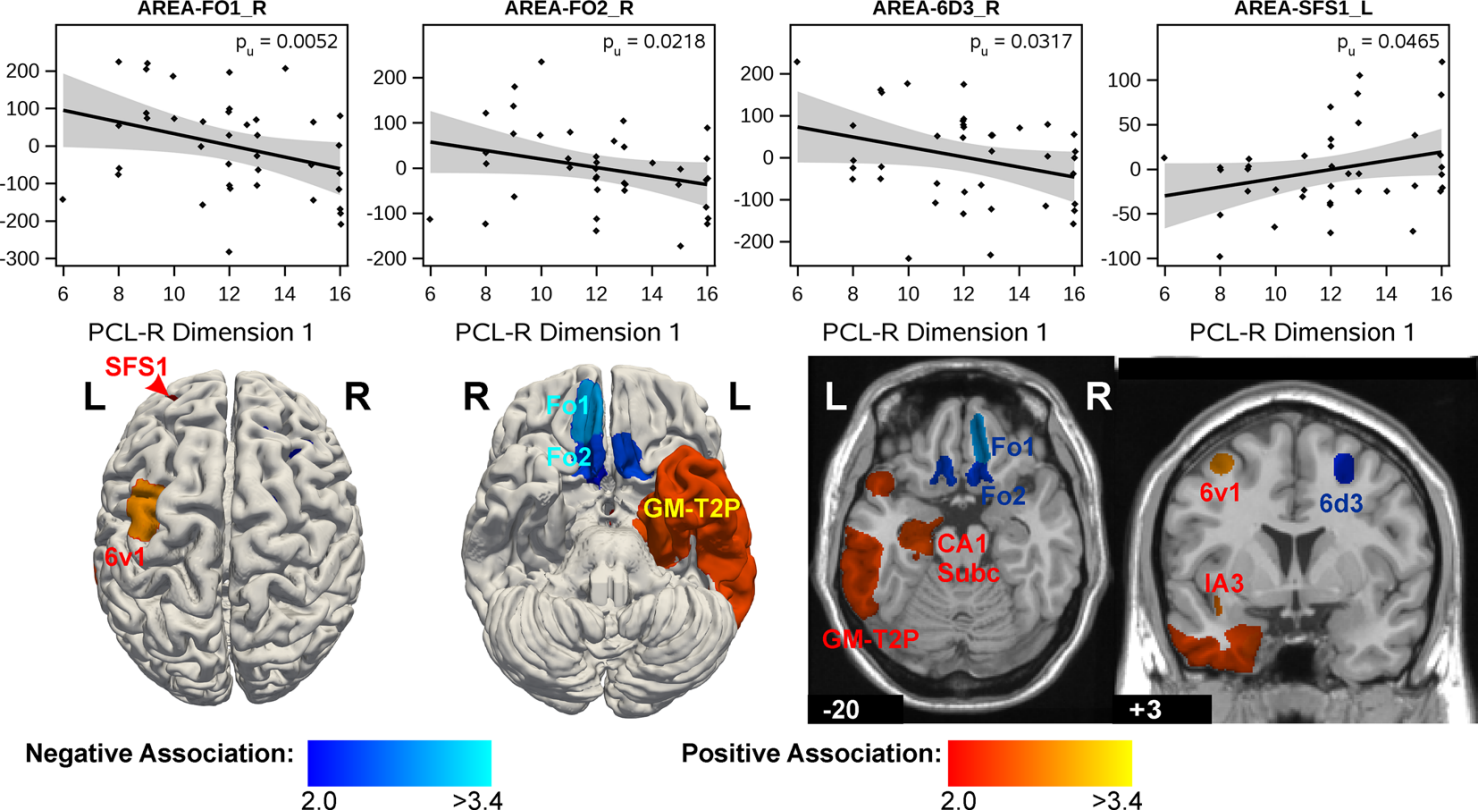
Associations of PCL-R factor 1 (inter-personal and affective features) with volumes of brain regions in psychopathic subjects with PCL-R total ≥ 20. Volume data were adjusted for study (acquisition site), age, ICV and PCL-R factor 2. The scatter-plots in the upper row show the individual data points, the regression line with its confidence limits and the p-value (uncorr.). The bottom row shows the brain regions, where an association with p_uncorr_ < 0.5 had been found, with colors corresponding to the statistical score (student’s t)

The associations of psychpopathic subjects’ brain morphology with PCL-R factor 1 revealed only few weak results. Negative associations with an uncorrected p-value ≤ 0.05 (i.e. reduced region volumes with increasing values of factor 1) had areas Fo1 and Fo2 in the medial OFC, and right dorsolateral frontal areas 8v2, 8d2 and premotor area 6d3. Areas with positive associations were located in the left hemisphere: Entorhinal cortex (EC), hippocampal area CA1 and prosubiculum (Hippocampus-Subc.ProS), piriform cortex area PirT.Tu, Gapmap-Temporal-to-Parietal and dorsal frontal area SFS 1 and premotor area 6v1, among other regions. However, none of these regions met the multiple-testing criterion. In particular no global effects were found. These results are shown in Fig. 1 and in Supplementary Table S1.

#### Associations of region volumes with PCL-R factor 2 (impulsive and antisocial behavior)

**Fig. 2:**
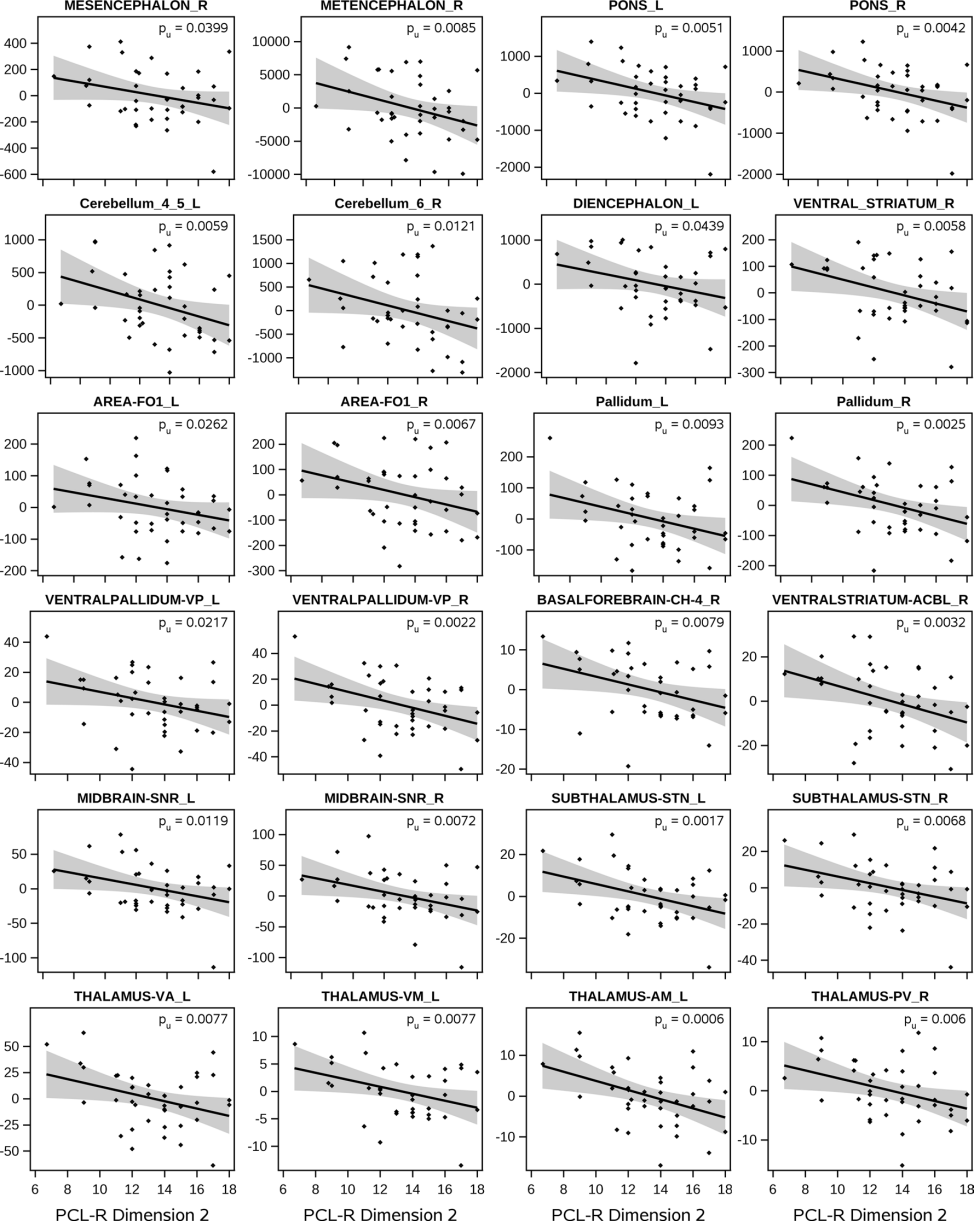
Associations of PCL-R factor 2 (deviant lifestyle and antisocial behavior) with volumes of brain regions in psychopathic subjects with PCL-R total ≥ 20. Volume data were adjusted for study (acquisition site), age, intra-cranial volume and PCL-R factor 1. The scatter-plots show the individual data points, the regression line with its confidence limits and the p-value (uncorr.)

**Fig. 3:**
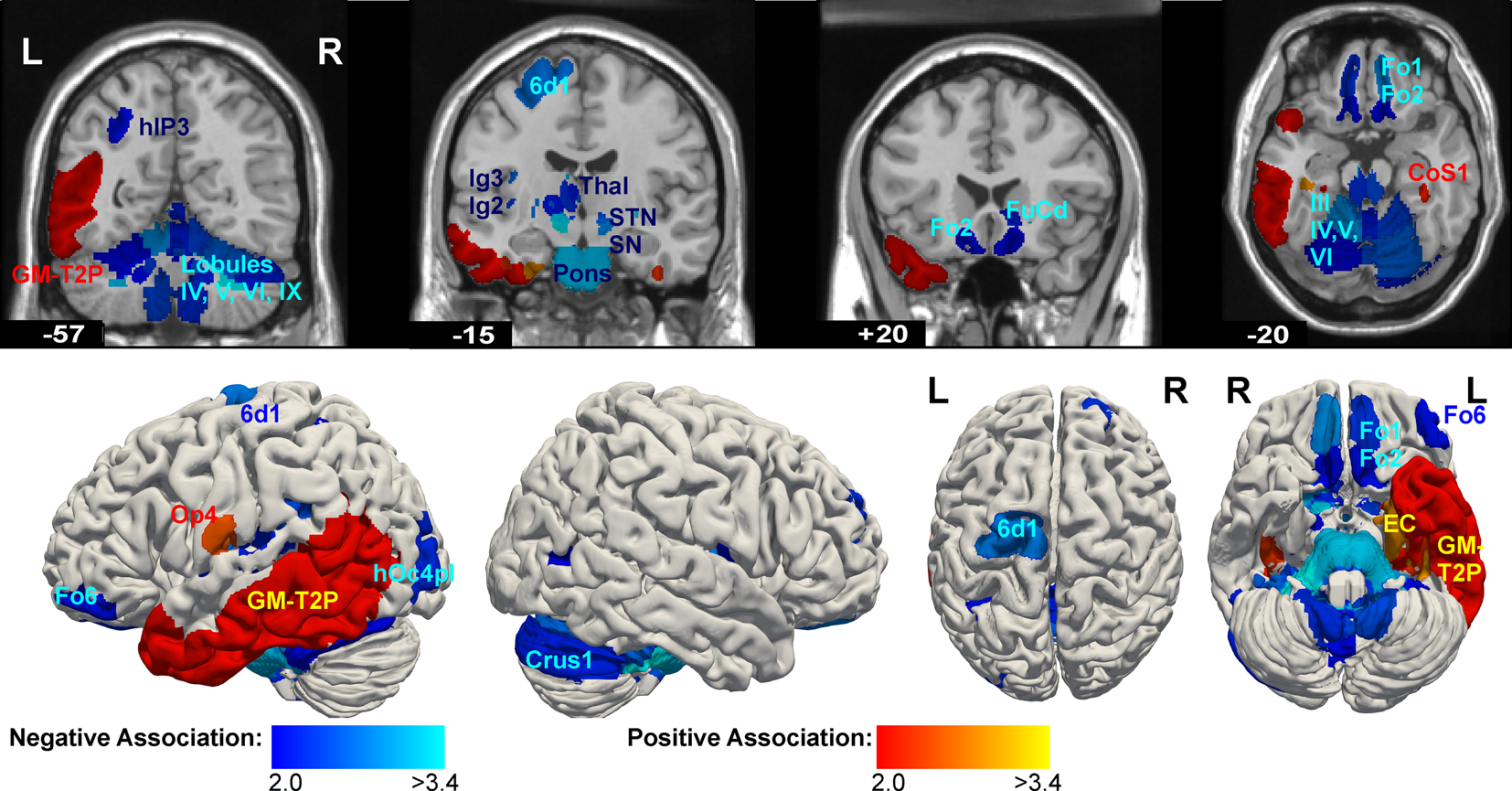
Associations of PCL-R factor 2 (deviant lifestyle and antisocial behavior) with volumes of brain regions in psychopathic subjects with PCL-R total ≥ 20. Volume data were adjusted for study (acquisition site), age, intra-cranial volume and PCL-R factor 1. Brain regions are shown, where an association with p_uncorr_ < 0.5 had been found, with colors corresponding to the statistical score (student’s t)

PCL-R factor 2 had predominantly negative associations with volumes of cortical areas and subcortical nuclei (Figs. 2 and 3, Supplementary Table S2). Particularly strong effects were found in the thalamus (e.g. anterior nuclei AM, AV, medial nucleus PV, ventral nuclei VA, VAMC, VLA, VM, among others), bilateral subthalamic nucleus (STN), mesencephalon (substantia nigra, red nucleus), and pons. Most of these effects were found bilaterally, and the effects in the STN, left anterior thalamus, mesencephalon and pons fulfilled the multiple-testing criterion. Further nuclei with negative associations were the pallidum (including the ventral pallidum VP) and the ventral striatum (nuclei AcbL, FuCd, FuP), mainly in the right hemisphere. Cortical areas having negative assocations with factor 2 were the medial orbitofrontal areas Fo1, Fo2 (bilaterally), Fo6 (right lateral OFC), dorsolateral frontal areas 6d1 (left), SFG3 and SFS1 (right). In the insular cortex, dysgranular areas Id4, Id5 and granular areas Ig1-3 had negative associations, as well as areas in the lateral occipital cortex, and left inferior parietal cortex, among other regions. In the cerebellum, bilateral cortical lobules, bilateral dentate nucleus, right interposed and fastigal nuclei showed negative associations. In contrast, left area EC (entorhinal cortex), piriform cortex areas PirT.Tit and Pir.TU, and bilateral areas Cos1 (collateral sulcus) and FG5 (fusiform gyrus) showed positive associations with factor 2.

#### Associations of region volumes with PCL-R total score

The PCL-R total score showed moderate to strong negative associations with the bilateral medial orbitofrontal areas Fo1 and Fo2 (Supplementary Table S3 and Figure S4). Further regions were the right dorsal and dorsolateral frontal areas 6ma, 8d2 and SFG3, insular area Id4, and left inferior parietal area PFcm, right ventral striatum nucleus AcbL, and few cerebellar lobules. Positive associations were found in the left area EC and piriform cortex areas PirT.Tit and Pir.TU, right hippocampal area CA1, left area CoS1 (collateral sulcus), and parietal area hPO1. However, none of these findings fulfilled the multiple-testing criterion. No effects were found on the global level.

### Group comparison of brain morphology between psychopathic subjects and controls

**Fig. 4:**
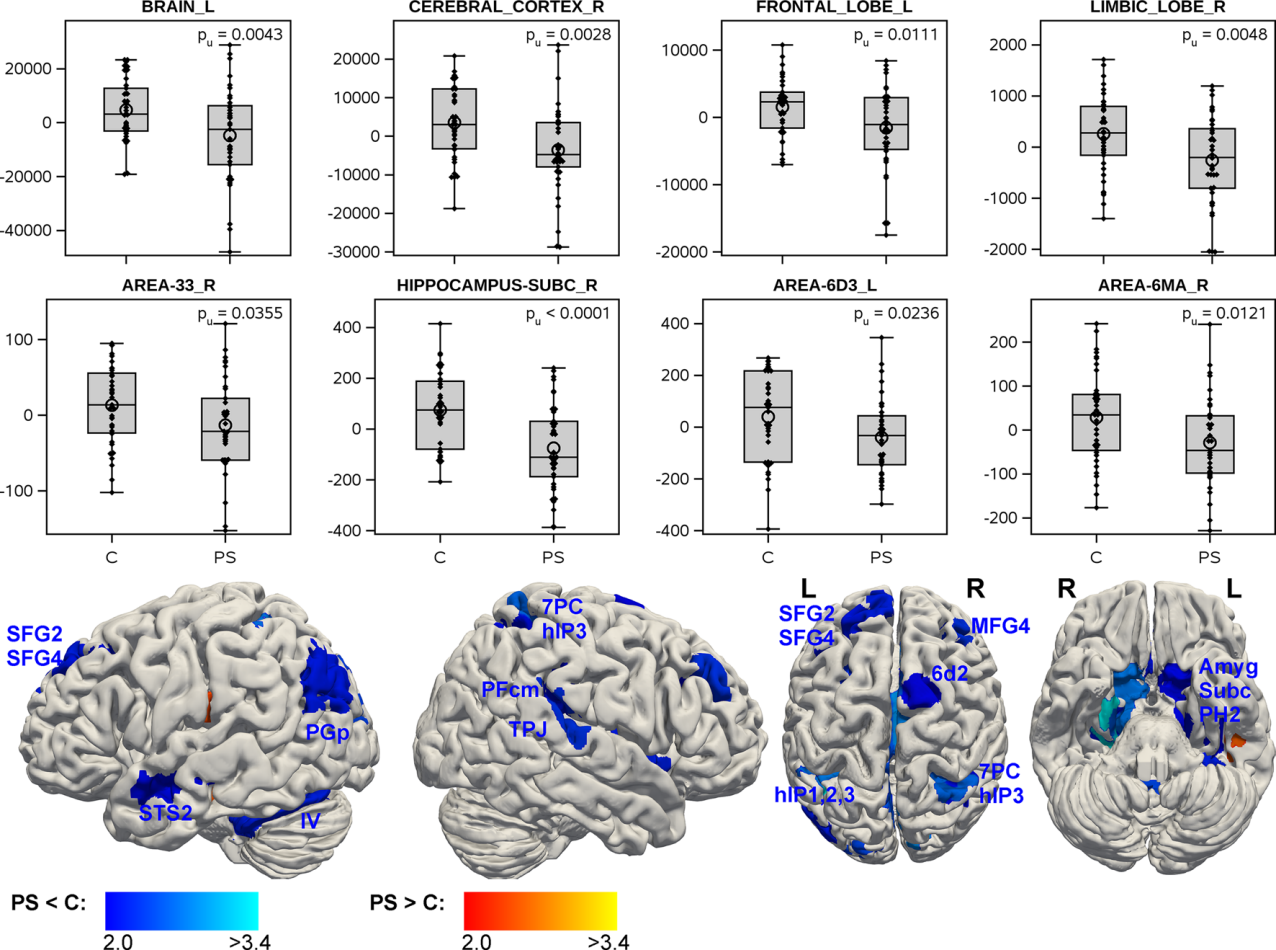
Group differences of brain region volumes between psychopathic subjects with PCL-R total ≥ 20 (PS) and healthy controls (C). Volume data were adjusted for study (acquisition site), age, and intra-cranial volume. The box-plots show the individual data points, the quantils of their distribution and the p-value (uncorr.). The bottom row shows the brain regions, where a group difference with p_uncorr_ < 0.5 had been found, with colors corresponding to the statistical score (student’s t)

The regional analysis revealed a significant volume deficit of the whole brain in psychopathic subjects (-1.45%), with similar effects in both hemispheres (left: -1.50%, right: -1.40%). In addition to these global effects, the right telencephalon and cerebral cortex showed volume deficits, which fulfilled the multiple-testing criterion (Supplementary Table S5). Moreover, several regions mainly in the cerebrum showed volume deficits in psychopathic subjects with p_uncorr_ < 0.05, however, without fulfilling the multiple-testing criterion. Relatively strong deficits were found in the right subiculum, besides right hippocampal area CA1 and cingulate area 33, as well as the left presubiclum. Further areas with volume deficits were located in the dorsal and dorsolateral frontal, parietal and temporal cortices of both hemispheres, in the left occipital cortex, right insula, and left cerebellum (Fig. 4, Supplementary Table S5).

## Discussion

### Associations of region volumes with PCL-R scores

The PCL-R factor 1 showed tendencies of both, positive and negative associations with brain region volumes. Regions in the bilateral medial orbitofrontal cortex and the right dorsolateral frontal cortex showed negative associations. Positive associations were found in the left thalamus, insula, hippocampal formation, dorsal frontal and temporal cortices and amygdala. Altough these findings didn’t meet the multiple testing criterion, they could support two conclusions: (1) The occurrence of positive assocations with PCL-R factor 1 in many brain regions is in contrast to factor 2, which had predominantly negative associations in our study. This could suggest that at least some of the behavioral features that are captured by factor 1 should *not* be interpreted as expressions of damages or dysfunctions of the brain. Indeed, the interpersonal-affective features which form PCL-R factor 1 characterise a cold-hearted, unemotional, unconscionable but also planned and instrumental, manipulative behavior. These features signify also so-called “successful psychopaths”, i.e. people who may act ruthlessly but not overtly criminally and who had been described as having relative high scores on the inter-personal scale, but at the same time relatively low anti-sociality scores [65]. Moreover, behavioral studies had shown, that factor 1 was negatively related to stress reactivity, and positively related to social dominance, higher adaptive functioning, socioenconomic status and verbal intelligence [65–67]. (2) The rather low statistical strenghts of the regional effects could indicate, that the behavioral features of factor 1 involve different circuits or regions in the brain. Interindividual differences in their involvement, however, might not be reflected by a one-dimensional score, thus requiring more specific behavioral characterisations.

A negative association between PCL-R factor 1 and GMV in medial orbitofrontal areas was reported by several previous studies but the associations with other regions were less consistent [18, 21, 22, 24, 29]. Further functional implications of the negative associations with the OFC are discussed below with regard to PCL-R factor 2.

The negative associations of factor 2 (impulsive and antisocial behavior) with region volumes in the bilateral mesencephalon (red nucleus, substantia nigra), pons, thalamus, subthalamic nucleus, pallidum, orbitofrontal areas Fo1, Fo2 and other areas in dorsal and lateral frontal cortices suggest a dysfunction of specific cortico-basal ganglia-thalamo-cortical circuits [68], which in brief form loops from areas in particular in the frontal cortex via basal ganglia structures (striatum, globus pallidus, subthalamic nucleus) and thalamic nuclei VA, VL, MD and CM back to the cortex. Disturbances of these circuits were found to be pertinent to the development of psychiatric disorders [69], and in particular impulsivity, tactlessness and irritability are described as neuropsychiatric consequences of dysfunctions of the orbitofrontal circuit [70]. Two fMRI-studies further support the role of these circuits in psychopathy, which found a negative association between the hemodynamic response in different basal ganglia structures and the psychopathy score of adolescent offenders in a response-inhibition paradigm [71], and aberrant effective connectivity between striatum and inferior frontal gyrus in conduct disorder patients when performing an inhibition control task [72]. The involvement of medial and dorsolateral prefrontal, orbitofronal, cingulate and insular cortices in aggression independently from psychopathy was shown in [73]. Reports of reduced response inhibition [74] or increased aggressive, impulsive behavior after deep brain stimulation in the subthalamic nucleus [75, 76] further support this assumption.

Previous morphometry studies reported heterogenous associations of PCL-R factor 2 with GMV: Negative associations had been found mainly in occipital and cingulate areas [36], in orbitofrontal cortex and temporal pole [20], with the prefrontal cortex volume [18] or amygdala volume [77]. Positive associations had been found in the basal ganglia, insula and orbitofrontal cortex [24] or in frontal, parietal, temporal cortices and cerebellum [21]. One study didn’t detect any associations with factor 2 [22]. A meta-analysis [29] of morphometrical studies reported negative associations with GMV in nearly all cerebral lobes and caudate nucleus. Despite the differences among these studies, there seems to be a tendency for essentially negative associations of factor 2 with brain structures, similar to our findings, even though we found associations primarily in subcortical regions and brainstem, besides cerebral cortex areas.

Most of the regions which showed in our study volumetric associations with the PCL-R total score showed also associations with PCL-R factor 1 or factor 2. To note are the negative associations of areas Fo1 and Fo2 in the bilateral medial orbitofrontal cortex. However, the regression analysis of factor 2 appeared to be more sensitive than that of the total score. This indicates a clear advantage of using more specific PCL-R factors instead of the PCL-R total score for the analysis of associations between brain structure and psychopathy.

### Group differences

The analysis of group differences between psychopathic subjects and controls demonstrated that the global brain volume of psychopathic subjects was diminished, and these differences could be attributed in particular to the cereberal cortex. Whereas these differences fulfilled the multiple-testing criterion, the regional effects on more detailed levels didn’t. Thus, despite the significant global grey matter deficit, pronounced regional focuses of volume deficits didn’t appear, suggesting a considerable inter-individual variability in the spatial spreading of volume losses. A tentative interpretation is that a disturbance of brain development with an inter-individually heterogenous spatial or regional pattern had occurred in the examined psychopathic subjects, with the individual regional pattern of these disturbances (besides other, e.g. environmental impacts) influencing the behavioral phenotype, as measured by the PCL-R scale. This assumption could also explain why group differences reported by different studies are also heterogenous in their spatial patterns [14, 15, 17]. Notwithstanding, the volume deficits in dorsolateral frontal regions, amygdala, hippocampal formation, anterior cingulate cortex and insular cortex are similar to findings in previous structural MRI studies [14, 16, 21, 77], and functional MRI studies likewise found activation differences between psychopathic subjects and controls in these regions [78]. These paralimbic and limbic regions are known to be highly engaged in social information processing, emotion regulation, attention and intrinsic control, and deficiencies in these areas often explain behavioral dysfunctions, aggression, impulsiveness, lack of empathy, and shallow affect, which are typical psychopathic traits [79].

Without going into detail, we mention two further theories [80–82] which postulate that the circuitry enganged in learning processes underlying socialization, moral decision-making, but also control of reactive aggression could be impaired in psychopathy. This circuitry encompasses in particular medial OFC, the amygdala but also the periaqueductal grey matter in the brainstem, which resemble the regions where we found either negative associations with factor 2 or volume deficits in the group comparison. Together with our findings these could demonstrate how multiple circuits might be involved in psychopathy.

### Strengths and limitations of this study

The combination of three samples increased the statistical power of our analysis. Another advantage was the application of a sensitive method to detect volume differences of small neuroanatomical regions. The use of different MR scanners and sequences at each site might influence the analysis, but the subject groups of each sample were matched. Therefore, this effect could be modelled by a categorical factor in the statistical analyses. A limitation of this study, however, is that results may also be influenced by cognitive capacity (e.g., intelligence, not quantified in all groups) or substance use. Ideally, these characteristics should be matched between psychopathic subjects and controls. However, while these factors may have influenced the group comparison, the correlation analyses are likely less affected, because these were calculated within the psychopathic group only, and we assume that differences between subjects in IQ or substance use were smaller *within* this group than between psychopathic subjects and controls.

## Conclusions

The present results suggest that the behavioral disturbances that are captured by the PCL-R factor 2 are associated with volume deficits in regions which belong to frontal-subcortical circuits that could be involved in behavioral control. The neurobiological substrates of the behavioral traits underlying PCL-R factor 1 appear to be more diverse, suggesting that more specific behavioral assessments might be necessary for their detection. The results of the group comparison tentatively suggest a rather widespread disturbance of brain development in psychopathic subjects. Questions for future studies are e.g., to what degree these structural differences are heritable or associated with e.g. environmental factors.

## Electronic supplementary material

Below is the link to the electronic supplementary material.

Supplementary Material 1

## Data Availability

Imaging data must not be provided because of regulations of data privacy protection.

## References

[1] Glenn AL, Raine A (2008) The neurobiology of psychopathy. Psychiatr Clin North Am 31:463–475. 10.1016/j.psc.2008.03.004 18638646 10.1016/j.psc.2008.03.004

[2] Reidy DE, Kearns MC, DeGue S, Lilienfeld SO, Massetti G, Kiehl KA (2015) Why psychopathy matters: implications for public health and violence prevention. Aggress Violent Beh 24:214–225. 10.1016/j.avb.2015.05.018 10.1016/j.avb.2015.05.018 PMC5868481 29593448

[3] Tharshini NK, Ibrahim F, Kamaluddin MR, Rathakrishnan B, Che Mohd Nasir N (2021) The link between individual personality traits and criminality: A systematic review. Int J Environ Res Public Health 18 10.3390/ijerph18168663 PMC8391956 34444412

[4] De Brito SA, Forth AE, Baskin-Sommers AR et al (2021) Psychopathy. Nat Reviews Disease Primers 7:49. 10.1038/s41572-021-00282-1 34238935 10.1038/s41572-021-00282-1

[5] Hare RD (1991) The Hare Psychopathy Checklist - Revised (PCL-R). 2nd. ed. edn

[6] Hare RD (2003) The Hare Psychopathy Checklist - Revised (PCL-R). 2nd. ed. edn

[7] Rodre S, Hedlund J, Liljeberg J, Kristiansson M, Masterman T, Sturup J (2019) Psychopathy-associated personality traits influence crime-scene behavior in male homicide offenders. Nord J Psychiatry 73:471–474. 10.1080/08039488.2019.1626907 31509039 10.1080/08039488.2019.1626907

[8] Declercq F, Willemsen J, Audenaert K, Verhaeghe P (2012) Psychopathy and predatory violence in homicide, violent, and sexual offences: factor and facet relations. Legal Criminological Psychol 17:59–74. 10.1348/135532510X527722

[9] Patrick CJ (1994) Emotion and psychopathy: startling new insights. Psychophysiology 31:319–330. 10.1111/j.1469-8986.1994.tb02440.x 10690912 10.1111/j.1469-8986.1994.tb02440.x

[10] Hare RD (2016) Psychopathy, the PCL-R, and criminal justice: some new findings and current issues. Can Psychol / Psychologie Canadienne 57:21–34. 10.1037/cap0000041

[11] Jeandarme I, Edens JF, Habets P, Bruckers L, Oei K, Bogaerts S (2017) PCL-R field validity in prison and hospital settings. Law Hum Behav 41:29–43. 10.1037/lhb0000222 27936826 10.1037/lhb0000222

[12] Weber S, Habel U, Amunts K, Schneider F (2008) Structural brain abnormalities in psychopaths—a review. Behav Sci Law 26:7–28. 10.1002/bsl.802 18327824 10.1002/bsl.802

[13] Koenigs M, Baskin-Sommers A, Zeier J, Newman JP (2011) Investigating the neural correlates of psychopathy: a critical review. Mol Psychiatry 16:792–799. 10.1038/mp.2010.124 21135855 10.1038/mp.2010.124 PMC3120921

[14] Pujol J, Harrison BJ, Contreras-Rodriguez O, Cardoner N (2019) The contribution of brain imaging to the Understanding of psychopathy. Psychol Med 49:20–31. 10.1017/S0033291718002507 30207255 10.1017/S0033291718002507

[15] Griffiths SY, Jalava JV (2017) A comprehensive neuroimaging review of PCL-R defined psychopathy. Aggress Violent Beh 36:60–75. 10.1016/j.avb.2017.07.002

[16] Johanson M, Vaurio O, Tiihonen J, Lähteenvuo M (2020) A systematic literature review of neuroimaging of psychopathic traits. Front Psychiatry 10. https://www.frontiersin.org/journals/psychiatry/articles/10.3389/fpsyt.2019.01027 10.3389/fpsyt.2019.01027 PMC7016047 32116828

[17] Deming P, Griffiths S, Jalava J, Koenigs M, Larsen RR (2024) Psychopathy and medial frontal cortex: A systematic review reveals predominantly null relationships. Neurosci Biobehavioral Reviews 167:105904. 10.1016/j.neubiorev.2024.105904 10.1016/j.neubiorev.2024.105904 PMC12208374 39343080

[18] Yang Y, Raine A, Lencz T, Bihrle S, LaCasse L, Colletti P (2005) Volume reduction in prefrontal Gray matter in unsuccessful criminal psychopaths. Biol Psychiatry 57:1103–1108. 10.1016/j.biopsych.2005.01.021 15866549 10.1016/j.biopsych.2005.01.021

[19] Yang Y, Raine A, Colletti P, Toga AW, Narr KL (2010) Morphological alterations in the prefrontal cortex and the amygdala in unsuccessful psychopaths. J Abnorm Psychol 119:546–554. 10.1037/a0019611 20677843 10.1037/a0019611

[20] Ermer E, Cope LM, Nyalakanti PK, Calhoun VD, Kiehl KA (2012) Aberrant paralimbic Gray matter in criminal psychopathy. J Abnorm Psychol 121:649–658. 10.1037/a0026371 22149911 10.1037/a0026371 PMC4039408

[21] Contreras-Rodríguez O, Pujol J, Batalla I et al (2015) Functional connectivity Bias in the prefrontal cortex of psychopaths. Biol Psychiatry 78:647–655. 10.1016/j.biopsych.2014.03.007 24742618 10.1016/j.biopsych.2014.03.007

[22] de Oliveira-Souza R, Hare RD, Bramati IE, Garrido GJ, Azevedo Ignácio F, Tovar-Moll F, Moll J (2008) Psychopathy as a disorder of the moral brain: Fronto-temporo-limbic grey matter reductions demonstrated by voxel-based morphometry. NeuroImage 40:1202–1213. 10.1016/j.neuroimage.2007.12.054 18289882 10.1016/j.neuroimage.2007.12.054

[23] Korponay C, Pujara M, Deming P et al (2017) Impulsive-antisocial psychopathic traits linked to increased volume and functional connectivity within prefrontal cortex. Soc Cognit Affect Neurosci 12:1169–1178. 10.1093/scan/nsx042 28402565 10.1093/scan/nsx042 PMC5490676

[24] Leutgeb V, Leitner M, Wabnegger A, Klug D, Scharmüller W, Zussner T, Schienle A (2015) Brain abnormalities in high-risk violent offenders and their association with psychopathic traits and criminal recidivism. Neuroscience 308:194–201. 10.1016/j.neuroscience.2015.09.011 26362887 10.1016/j.neuroscience.2015.09.011

[25] Korponay C, Dentico D, Kral T et al (2017) Neurobiological correlates of impulsivity in healthy adults: lower prefrontal Gray matter volume and spontaneous eye-blink rate but greater resting-state functional connectivity in basal ganglia-thalamo-cortical circuitry. NeuroImage 157:288–296. 10.1016/j.neuroimage.2017.06.015 28602816 10.1016/j.neuroimage.2017.06.015 PMC5600835

[26] Müller JL, Gänßbauer S, Sommer M, Döhnel K, Weber T, Schmidt-Wilcke T, Hajak G (2008) Gray matter changes in right superior Temporal gyrus in criminal psychopaths. Evidence from voxel-based morphometry. Psychiatry Research: Neuroimaging 163:213–222. 10.1016/j.pscychresns.2007.08.010 10.1016/j.pscychresns.2007.08.010 18662867

[27] Laakso MP, Vaurio O, Koivisto E et al (2001) Psychopathy and the posterior hippocampus. Behav Brain Res 118:187–193. 10.1016/S0166-4328(00)00324-7 11164516 10.1016/s0166-4328(00)00324-7

[28] González-Alemañy E, Rodríguez Olivera AD, Bobes MA, Armony JL (2023) Brain structural correlates of psychopathic traits in elite female combat-sports athletes. Eur J Neurosci 58:4255–4263. 10.1111/ejn.16171 37884281 10.1111/ejn.16171

[29] De Brito SA, McDonald D, Camilleri JA, Rogers JC (2021) Cortical and subcortical Gray matter volume in psychopathy: A voxel-wise meta-analysis. J Abnorm Psychol 130:627–640. 10.1037/abn0000698 34553958 10.1037/abn0000698

[30] Ashburner J, Friston KJ (2000) Voxel-based morphometry–the methods. NeuroImage 11:805–821. 10.1006/nimg.2000.0582 10860804 10.1006/nimg.2000.0582

[31] Gregory S, ffytche D, Simmons A, Kumari V, Howard M, Hodgins S, Blackwood N (2012) The antisocial brain: psychopathy matters: A structural MRI investigation of antisocial male violent offenders. Arch Gen Psychiatry 69:962–972. 10.1001/archgenpsychiatry.2012.222 22566562 10.1001/archgenpsychiatry.2012.222

[32] Korponay C, Pujara M, Deming P et al (2017) Impulsive-Antisocial dimension of psychopathy linked to enlargement and abnormal functional connectivity of the striatum. Biol Psychiatry: Cogn Neurosci Neuroimaging 2:149–157. 10.1016/j.bpsc.2016.07.004 28367514 10.1016/j.bpsc.2016.07.004 PMC5373097

[33] Tiihonen J, Rossi R, Laakso MP et al (2008) Brain anatomy of persistent violent offenders: more rather than less. Psychiatry Research: Neuroimaging 163:201–212. 10.1016/j.pscychresns.2007.08.012 10.1016/j.pscychresns.2007.08.012 18662866

[34] Bertsch K, Grothe M, Prehn K et al (2013) Brain volumes differ between diagnostic groups of violent criminal offenders. Eur Arch Psychiatry Clin NeuroSci 263:593–606. 10.1007/s00406-013-0391-6 23381548 10.1007/s00406-013-0391-6

[35] Cope LM, Shane MS, Segall JM et al (2012) Examining the effect of psychopathic traits on Gray matter volume in a community substance abuse sample. Psychiatry Research: Neuroimaging 204:91–100. 10.1016/j.pscychresns.2012.10.004 10.1016/j.pscychresns.2012.10.004 PMC3536442 23217577

[36] Calzada-Reyes A, Alvarez-Amador A, Galán-Garcia L, Valdés-Sosa M (2021) Electroencephalographic and morphometric abnormalities in psychopath offenders. Behav Sci Law 39:597–610. 10.1002/bsl.2548 34800344 10.1002/bsl.2548

[37] Puri BK, Counsell SJ, Saeed N, Bustos MG, Treasaden IH, Bydder GM (2008) Regional grey matter volumetric changes in forensic schizophrenia patients: an MRI study comparing the brain structure of patients who have seriously and violently offended with that of patients who have not. BMC Psychiatry 8:S6. 10.1186/1471-244X-8-S1-S6 18433516 10.1186/1471-244X-8-S1-S6 PMC2330074

[38] Hofhansel L, Weidler C, Votinov M, Clemens B, Raine A, Habel U (2020) Morphology of the criminal brain: Gray matter reductions are linked to antisocial behavior in offenders. Brain Struct Function 225:2017–2028. 10.1007/s00429-020-02106-6 10.1007/s00429-020-02106-6 PMC7473962 32591929

[39] Schneider F, Habel U, Kessler C, Posse S, Grodd W, Müller-Gärtner H-W (2000) Functional imaging of conditioned aversive emotional responses in antisocial personality disorder. Neuropsychobiology 42:192–201. 10.1159/000026693 11096335 10.1159/000026693

[40] Amunts K, Mohlberg H, Bludau S, Zilles K (2020) Julich-Brain: A 3D probabilistic atlas of the human brain’s cytoarchitecture. Science 369:988–992. 10.1126/science.abb4588 32732281 10.1126/science.abb4588

[41] Babiak P, Neumann CS, Hare RD (2010) Corporate psychopathy: talking the walk. Behav Sci Law 28:174–193. 10.1002/bsl.925 20422644 10.1002/bsl.925

[42] Wittchen H-U, Gruschwitz S, Wunderlich U, Zaudig M (1997) Strukturiertes klinisches interview für DSM-IV (SKID-I). Achse I: Psychische störungen. Hogrefe, (Göttingen

[43] Mokros A, Hollerbach P, Vohs K, Nitschke J, Eher R, Habermeyer E (2013) Normative data for the psychopathy Checklist–Revised in German-Speaking countries: A Meta-Analysis. Criminal Justice Behav 40:1397–1412. 10.1177/0093854813492519

[44] Hare RD, Hart SD, Harpur TJ (1991) Psychopathy and the DSM-IV criteria for antisocial personality disorder. J Abnorm Psychol 100:391–398. 10.1037/0021-843X.100.3.391 1918618 10.1037//0021-843x.100.3.391

[45] Hare RD, Clark D, Grann M, Thornton D (2000) Psychopathy and the predictive validity of the PCL-R: an international perspective. Behav Sci Law 18:623–645. 10.1002/1099-0798(200010)18:5%3C623::AID-BSL409%3E3.0.CO;2-W 11113965 10.1002/1099-0798(200010)18:5<623::aid-bsl409>3.0.co;2-w

[46] Hartmann J, Hollweg M, Nedopil N (2001) Quantitative erfassung dissozialer und psychopathischer persönlichkeiten Bei der Strafrechtlichen begutachtungretrospektive untersuchung Zur Anwendbarkeit der Deutschen version der Hare-Psychopathie-Checkliste. Nervenarzt 72:365–370. 10.1007/s001150050765 11386147 10.1007/s001150050765

[47] Hare RD (2003) The Hare Psychopathy Checklist - Revised (PCL-R): Technical Manual. 2nd ed. edn

[48] Pieperhoff P, Hömke L, Schneider F, Habel U, Shah NJ, Zilles K, Amunts K (2008) Deformation field morphometry reveals age-related structural differences between the brains of adults up to 51 years. J Neurosci 28:828–842. 10.1523/JNEUROSCI.3732-07.2008 18216191 10.1523/JNEUROSCI.3732-07.2008 PMC6671008

[49] Ashburner J, Friston KJ (2005) Unified segmentation. NeuroImage 26:839–851. 10.1016/j.neuroimage.2005.02.018 15955494 10.1016/j.neuroimage.2005.02.018

[50] Yushkevich PA, Piven J, Hazlett HC, Smith RG, Ho S, Gee JC, Gerig G (2006) User-guided 3D active contour segmentation of anatomical structures: significantly improved efficiency and reliability. NeuroImage 31:1116–1128. 10.1016/j.neuroimage.2006.01.015 16545965 10.1016/j.neuroimage.2006.01.015

[51] Holmes CJ, Hoge R, Collins L, Woods R, Toga AW, Evans AC (1998) Enhancement of MR images using registration for signal averaging. J Comput Assist Tomogr 22:324–333 9530404 10.1097/00004728-199803000-00032

[52] Jenkinson M, Smith S (2001) A global optimisation method for robust affine registration of brain images. Med Image Anal 5:143–156. 10.1016/S1361-8415(01)00036-6 11516708 10.1016/s1361-8415(01)00036-6

[53] Krause D, Thörnig P (2016) JURECA: General-purpose supercomputer at Jülich supercomputing centre. JLSRF 2. 10.17815/jlsrf-2-121

[54] Rolls ET, Huang C-C, Lin C-P, Feng J, Joliot M (2020) Automated anatomical labelling atlas 3. NeuroImage 206:116189. 10.1016/j.neuroimage.2019.116189 31521825 10.1016/j.neuroimage.2019.116189

[55] Tzourio-Mazoyer N, Landeau B, Papathanassiou D et al (2002) Automated anatomical labeling of activations in SPM using a macroscopic anatomical parcellation of the MNI MRI single-subject brain. NeuroImage 15:273–289. 10.1006/nimg.2001.0978 11771995 10.1006/nimg.2001.0978

[56] Lynam DR, Hoyle RH, Newman JP (2006) The perils of partialling: cautionary Tales from aggression and psychopathy. Assessment 13:328–341. 10.1177/1073191106290562 16880283 10.1177/1073191106290562 PMC3152746

[57] Yekutieli D (2008) Hierarchical false discovery Rate–Controlling methodology. J Am Stat Assoc 103:309–316. 10.1198/016214507000001373

[58] Bogomolov M, Peterson CB, Benjamini Y, Sabatti C (2020) Hypotheses on a tree: new error rates and testing strategies. Biometrika 108:575–590. 10.1093/biomet/asaa086 36825068 10.1093/biomet/asaa086 PMC9945647

[59] Barber RF, Ramdas A (2016) The p-filter: multilayer false discovery rate control for grouped hypotheses. J Royal Stat Soc Ser B: Stat Methodol 79:1247–1268. 10.1111/rssb.12218

[60] Simes RJ (1986) An improved Bonferroni procedure for multiple tests of significance. Biometrika 73:751–754. 10.1093/biomet/73.3.751

[61] Heller R, Stanley D, Yekutieli D, Rubin N, Benjamini Y (2006) Cluster-based analysis of FMRI data. NeuroImage 33:599–608. 10.1016/j.neuroimage.2006.04.233 16952467 10.1016/j.neuroimage.2006.04.233

[62] Benjamini Y, Hochberg Y (1995) Controlling the false discovery rate: A practical and powerful approach to multiple testing. J Roy Stat Soc: Ser B (Methodol) 57:289–300. 10.1111/j.2517-6161.1995.tb02031.x

[63] Benjamini Y, Heller R (2007) False discovery rates for Spatial signals. J Am Stat Assoc 102:1272–1281. 10.1198/016214507000000941

[64] Hare RD, Harpur TJ, Hakstian AR, Forth AE, Hart SD, Newman JP (1990) The revised psychopathy checklist: reliability and factor structure. Psychol Assessment: J Consulting Clin Psychol 2:338–341. 10.1037/1040-3590.2.3.338

[65] Hall JR, Benning SD (2006) Guilford Press,. in Handbook of Psychopathy (ed Christopher J. Patrick) Ch. 23, 459–478

[66] Hall JR, Benning SD, Patrick CJ (2004) Criterion-Related validity of the Three-Factor model of psychopathy: personality, behavior, and adaptive functioning. Assessment 11:4–16. 10.1177/1073191103261466 14994949 10.1177/1073191103261466

[67] Vitacco MJ, Neumann CS, Jackson RL (2005) Testing a Four-Factor model of psychopathy and its association with ethnicity, gender, intelligence, and violence. J Consult Clin Psychol 73:466–476. 10.1037/0022-006X.73.3.466 15982144 10.1037/0022-006X.73.3.466

[68] Alexander GE, Crutcher MD (1990) Functional architecture of basal ganglia circuits: neural substrates of parallel processing. Trends Neurosci 13:266–271. http://www.sciencedirect.com/science/article/pii/016622369090107L 1695401 10.1016/0166-2236(90)90107-l

[69] Macpherson T, Hikida T (2019) Role of basal ganglia neurocircuitry in the pathology of psychiatric disorders. J Neuropsychiatry Clin Neurosci 73:289–301. 10.1111/pcn.12830 10.1111/pcn.12830 30734985

[70] Bonelli RM, Cummings JL (2007) Frontal-subcortical circuitry and behavior. Dialog Clin Neurosci 9:141–151. 10.31887/DCNS.2007.9.2/rbonelli 10.31887/DCNS.2007.9.2/rbonelli PMC3181854 17726913

[71] Maurer JM, Steele VR, Vincent GM, Rao V, Calhoun VD, Kiehl KA (2019) Adolescent psychopathic traits negatively relate to hemodynamic activity within the basal ganglia during Error-Related processing. J Abnorm Child Psychol 47:1917–1929. 10.1007/s10802-019-00560-3 31104203 10.1007/s10802-019-00560-3 PMC6842671

[72] Zhang J, Li B, Gao J et al (2015) Impaired Frontal-Basal ganglia connectivity in male adolescents with conduct disorder. PLoS ONE 10:e0145011. 10.1371/journal.pone.0145011 26658732 10.1371/journal.pone.0145011 PMC4682835

[73] Repple J, Pawliczek CM, Voss B, Siegel S, Schneider F, Kohn N, Habel U (2017) From provocation to aggression: the neural network. BMC Neurosci 18:73. 10.1186/s12868-017-0390-z 29041906 10.1186/s12868-017-0390-z PMC5646154

[74] Ballanger B, van Eimeren T, Moro E et al (2009) Stimulation of the subthalamic nucleus and impulsivity: release your horses. Ann Neurol 66:817–824. 10.1002/ana.21795 20035509 10.1002/ana.21795 PMC2972250

[75] Papuć E, Trojanowski T, Obszańska K, Stelmasiak Z (2015) Aggressive behavior as a rare side effect of subthalamic stimulation in Parkinson’s disease. Neurocase 21:220–225. 10.1080/13554794.2014.890729 24564255 10.1080/13554794.2014.890729

[76] Sensi M, Eleopra R, Cavallo MA et al (2004) Explosive-aggressive behavior related to bilateral subthalamic stimulation. Parkinsonism Relat Disord 10:247–251. 10.1016/j.parkreldis.2004.01.007 15120100 10.1016/j.parkreldis.2004.01.007

[77] Yang Y, Raine A, Narr KL, Colletti P, Toga AW (2009) Localization of deformations within the amygdala in individuals with psychopathy. Arch Gen Psychiatry 66:986–994. 10.1001/archgenpsychiatry.2009.110 19736355 10.1001/archgenpsychiatry.2009.110 PMC3192811

[78] Birbaumer N, Veit R, Lotze M, Erb M, Hermann C, Grodd W, Flor H (2005) Deficient fear conditioning in psychopathy: A functional magnetic resonance imaging study. Arch Gen Psychiatry 62:799–805. 10.1001/archpsyc.62.7.799 15997022 10.1001/archpsyc.62.7.799

[79] Kiehl KA (2006) A cognitive neuroscience perspective on psychopathy: evidence for paralimbic system dysfunction. Psychiatry Res 142:107–128. 10.1016/j.psychres.2005.09.013 16712954 10.1016/j.psychres.2005.09.013 PMC2765815

[80] Blair RJR (2007) Dysfunctions of medial and lateral orbitofrontal cortex in psychopathy. Ann N Y Acad Sci 1121:461–479. 10.1196/annals.1401.017 17698995 10.1196/annals.1401.017

[81] Blair RJR (2010) Psychopathy, frustration, and reactive aggression: the role of ventromedial prefrontal cortex. Br J Psychol 101:383–399. 10.1348/000712609X418480 19321035 10.1348/000712609X418480

[82] Raine A (2019) The neuromoral theory of antisocial, violent, and psychopathic behavior. Psychiatry Res 277:64–69. 10.1016/j.psychres.2018.11.025 30473129 10.1016/j.psychres.2018.11.025

