# Supplementary material for: Associations of brain structure with psychopathy
Source: Eur Arch Psychiatry Clin Neurosci. 2025 May 29;276(1):63–75. doi: 10.1007/s00406-025-02028-6 (PMC12904969; doi:10.1007/s00406-025-02028-6)
Supplement: Supplementary file 1 — Supplementary Material 1 [file 406_2025_2028_MOESM1_ESM.pdf]

Supplementary material of

Associations of brain structure with psychopathy

European Archives of Psychiatry and Clinical Neuroscience

Peter Pieperhoff[1], Lena Hofhansel[2,3], Frank Schneider[4], Jürgen Müller[6], Katrin Amunts[1,8], Sabrina Weber-Papen[5], Carmen Weidler[2], Benjamin Clemens[2], Adrian Raine [7], Ute Habel [2,3]

[1] Institute of Neuroscience and Medicine (INM-1), Research Centre Jülich, Jülich, Germany

[2] Department of Psychiatry, Psychotherapy and Psychosomatics, Medical Faculty, RWTH Aachen University, Aachen, Germany

[3] Institute of Neuroscience and Medicine (INM-10), Research Centre Jülich, Jülich, Germany

[4] Department of History, Philosophy and Ethics of Medicine, School of Medicine, Heinrich-Heine-University Düsseldorf, Germany

[5] University Hospital Düsseldorf, Heinrich Heine University Düsseldorf, Düsseldorf, Germany

[6] Department of Psychiatry, Forensic Psychiatry, Human Medical Center, Georg August University Göttingen, Germany

[7] Departments of Criminology, Psychiatry, and Psychology, University of Pennsylvania, Philadelphia, PA, USA

[8] C. and O. Vogt Institute for Brain Research, University Hospital Düsseldorf, Heinrich Heine University Düsseldorf, Düsseldorf, Germany

<u>Corresponding author:</u> Peter Pieperhoff (Email: p.pieperhoff@fz-juelich.de)

Methods

Magnetic resonance imaging

The following MR scanners and sequence parameters had been applied in each subsample:

(1) Data set MU (Müller et al. 2008) was recorded at the University Hospital in Göttingen, Germany with a 1.5 Tesla Siemens Symphony scanner (Siemens Medical Systems, Erlangen, Germany). T1-weighted images were acquired with a magnetization-prepared rapid acquisition gradient echo image (MPRAGE) sequence with a voxel size of 1 x 1 x 1.08 mm (matrix size 256 x 192, TR = 11.08 ms, TE = 4 ms, flip angle = 15°).

(2) Data set (FS, UH) (Schneider et al. 2000) was recorded at the Forschungszentrum Jülich with a 1.5 Tesla Magnetom Vision Scanner (Siemens Medical Systems, Erlangen, Germany). A MPRAGE sequence with a voxel size of 0.9 x 0.9 x 1.25 mm and 0.9 x 0.9 x1.33 mm was used (matrix size: 256 x 256, TR = 704, TE = 4.4 ms).

(3) Data set (UH, LH) (Hofhansel et al. 2020) was acquired using a 3 Tesla Siemens PRISMA MR scanner (Siemens Medical Systems, Erlangen, Germany), located in the Medical Faculty of RWTH Aachen University. T1-weighted structural images were obtained by means of a three-dimensional MPRAGE sequence (voxel size: 1 × 1 × 1 mm, matrix size: 256 x 256, TR = 2300ms, TE = 2.98ms, flip angle = 9°).

Measurement of the intracranial volume (ICV)

The intracranial volume (ICV) of each subject was measured by manual segmentation. First, the MR images were affinely transformed into the space of the reference brain so that the transformed brains had nearly the same extent and alignment. The contour of the intracranial vault was manually delineated on every tenth sagittal section, as well as on the three most lateral sections, using the program ITK-SNAP. The sizes of the segmented mask sections were calculated and linearly interpolated for sections in between. The summed section sizes yielded the ICV in the space of the reference brain, which was rescaled by the determinant of the affine transformation to yield the actual ICV.

Results of region-based analysis

The brain regions are arranged in a hierarchical atlas system with the whole brain on the highest level, and cortical areas and subcortical nuclei at the lowest level. The latter are from the Julich-Brain atlas (Amunts et al. 2020), complemented by maps of nuclei and cerebellar lobules of the AAL3 atlas (Tzourio-Mazoyer et al. 2002, Rolls et al. 2020), and masks of pons, mesencephalon and cerebellar white matter. Regions on upper levels are formed by combination of their child-regions. For every region within the hierarchy the test-statistic t_{uncorr} is calculated by evaluation of the respective statistical model with the region's volume data. The multiple-testing correction follows the procedure described in (Bogomolov et al. 2020).

All regions which have the same *parent region* form a *region family*. The p-value of such a family can be calculated by direct statistical evaluation of the parent region's volume data, which results in the uncorrected p-value p_{uncorr}. Alternatively, this p-value can be calculated by the Simes p-value (Simes 1986) of the family's member regions, see main text for details. Then within each family, the multiple-testing correction of the p-values of its members is calculated using the Benjamini-Hochberg method for controlling the false discovery rate (FDR)(Benjamini and Hochberg 1995), yielding the corrected p-value p_{fdr}. When p_{fdr} is below the specified FDR threshold, the multiple-testing criterion is fulfilled. However, according to (Bogomolov et al. 2020) the FDR threshold q_{set} has to be reduced by the proportion of "significant findings" or "discoveries" in the upper region. This procedure must only be applied within a region family, when its parent region was found to have a significant effect.

The following tables show the results of the atlas branches down to every region, where at least the uncorrected p-value is < 0.05. The column "sign" indicates columns with significant findings. Significance of multiple-testing corrected p-value: * p<0.05, ** p<0.01, *** p<0.001, **** p<0.0001.

u = uncorrected p-value ≤ 0.05 .

Table S1: Associations between PCL-R dimension 1 and regions of hierarchical brain atlas

REGION	t_uncorr	p_simes	p_fdr	sign	q_set
BRAIN	0.79	0.9730	0.9730		0.0500
├── BRAIN_L	0.78	0.5660	0.9730		0.0500
│ ├── DIENCEPHALON_L	1.12	0.1420	0.5660		0.0000
│ │ └── THALAMUS_L	1.09	0.4310	0.4310		0.0000
│ │ └── DORSAL_THALAMUS_L	1.03	0.2880	0.4310		0.0000
│ │ └── MEDIAL_GROUP_L	0.52	0.0957	0.2880		0.0000
│ │ └── THALAMUS-MV_L	2.24	0.0319	0.0957	u	0.0000
│ └── TELENCEPHALON_L	1.39	0.5510	0.7210		0.0000
│ ├── CEREBRAL_CORTEX_L	1.14	0.5510	0.5510		0.0000
│ │ ├── FRONTAL_LOBE_L	0.71	0.5710	0.6850		0.0000
│ │ │ ├── MEDIAL_ORBITOFRONTAL_CORTEX_L	-0.92	0.0594	0.6240		0.0000
│ │ │ │ └── AREA-FO2_L	-2.45	0.0198	0.0594	u	0.0000
│ │ │ ├── SUPERIOR_FRONTAL_SULCUS_L	1.90	0.1370	0.8870		0.0000
│ │ │ │ └── AREA-SFS1_L	2.07	0.0465	0.1370	u	0.0000
│ │ │ └── VENTRAL_PRECENTRAL_GYRUS_L	1.44	0.0272	0.5710	u	0.0000
│ │ │ └── AREA-6V1_L	2.89	0.0068	0.0272	u	0.0000
│ │ ├── INSULA_L	0.40	0.0918	0.5510		0.0000
│ │ │ └── AGRANULAR_INSULA_L	1.93	0.0306	0.0918	u	0.0000
│ │ │ └── AREA-IA3_L	2.73	0.0102	0.0306	u	0.0000
│ │ ├── LIMBIC_LOBE_L	1.29	0.3670	0.5510		0.0000
│ │ │ ├── HIPPOCAMPAL_FORMATION_L	2.50	0.1840	0.3670		0.0000
│ │ │ │ ├── AREA-EC_L	2.30	0.0283	0.1840	u	0.0000
│ │ │ │ ├── HIPPOCAMPUS-CA1_L	2.08	0.0459	0.1840	u	0.0000
│ │ │ │ └── HIPPOCAMPUS-SUBC_L	1.73	0.1040	0.2360		0.0000
│ │ │ │ └── HIPPOCAMPUS-SUBC.PROS_L	2.33	0.0261	0.1040	u	0.0000
│ │ │ └── PIRIFORM_CORTEX_L	1.99	0.1360	0.3670		0.0000
│ │ │ └── AREA-PIRT_L	2.10	0.0679	0.1360		0.0000
│ │ │ └── AREA-PIRT.TU_L	2.19	0.0358	0.0679	u	0.0000
│ │ └── TEMPORAL_LOBE_L	2.07	0.2220	0.5510		0.0000
│ │ └── GAPMAP-TEMPORAL-TO-PARIETAL_L	2.44	0.0202	0.2220	u	0.0000
│ └── CEREBRAL_NUCLEI_L	1.56	0.3380	0.5510		0.0000
│ └── AMYGDALA_L	1.69	0.4550	0.4550		0.0000
│ └── AMYGDALA-CM_L	1.99	0.0852	0.4550		0.0000
│ └── AMYGDALA-CM.AAA_L	2.30	0.0284	0.0852	u	0.0000
└── BRAIN_R	0.77	0.9730	0.9730		0.0500
└── TELENCEPHALON_R	1.14	0.9730	0.9730		0.0000
└── CEREBRAL_CORTEX_R	0.89	0.9730	0.9730		0.0000
└── FRONTAL_LOBE_R	-0.07	0.3280	0.9730		0.0000
├── MEDIAL_ORBITOFRONTAL_CORTEX_R	-1.76	0.0156	0.3280	u	0.0000
│ ├── AREA-FO1_R	-3.00	0.0052	0.0156	u	0.0000
│ └── AREA-FO2_R	-2.41	0.0218	0.0327	u	0.0000
├── MIDDLE_FRONTAL_GYRUS_R	-0.43	0.1670	0.9370		0.0000
│ └── AREA-8V2_R	-2.12	0.0418	0.1670	u	0.0000
├── SUPERIOR_FRONTAL_GYRUS_R	-1.27	0.1780	0.9370		0.0000
│ └── AREA-8D2_R	-2.09	0.0446	0.1780	u	0.0000
└── SUPERIOR_FRONTAL_SULCUS_R	-1.18	0.1270	0.9370		0.0000
└── AREA-6D3_R	-2.25	0.0317	0.1270	u	0.0000

Table S1: Associations between PCL-R dimension 1 and regions of the hierarchical brain atlas. p-values of higher-level regions where calculated using the Simes method (see above and main text).

Table S2: Associations between PCL-R dimension 2 and regions of hierarchical brain atlas

REGION	t_uncorr	p_simes	p_fdr	sign	q_set
BRAIN	-1.86	0.0240	0.0240	*	0.0500
├── BRAIN_L	-1.66	0.0136	0.0240	*	0.0500
│ ├── DIENCEPHALON_L	-2.10	0.0034	0.0136	**	0.0500
│ │ ├── SUBTHALAMUS_L	-3.44	0.0017	0.0034	**	0.0375
│ │ │ └── SUBTHALAMUS-STN_L	-3.44	0.0017	0.0017	**	0.0375
│ │ └── THALAMUS_L	-2.06	0.0135	0.0135	*	0.0375
│ │ ├── DORSAL_THALAMUS_L	-2.04	0.0090	0.0135	**	0.0375
│ │ │ ├── ANTERIOR_GROUP_L	-2.57	0.0018	0.0090	**	0.0250
│ │ │ │ ├── THALAMUS-AM_L	-3.82	0.0006	0.0018	***	0.0050
│ │ │ │ └── THALAMUS-AV_L	-3.25	0.0027	0.0041	**	0.0050
│ │ │ ├── INTRALAMINAR_GROUP_L	-2.19	0.0508	0.0635		0.0250
│ │ │ │ ├── THALAMUS-CL_L	-2.34	0.0254	0.0508	u	0.0050
│ │ │ │ └── THALAMUS-SPF_L	-2.46	0.0195	0.0508	u	0.0050
│ │ │ ├── MEDIAL_GROUP_L	-2.38	0.0408	0.0635	u	0.0250
│ │ │ │ ├── THALAMUS-MD_L	-2.17	0.0376	0.0564	u	0.0050
│ │ │ │ └── THALAMUS-PV_L	-2.61	0.0136	0.0408	u	0.0050
│ │ │ └── VENTRAL_GROUP_L	-2.40	0.0257	0.0635	u	0.0250
│ │ │ ├── THALAMUS-VAMC_L	-2.96	0.0057	0.0257	u	0.0050
│ │ │ ├── THALAMUS-VA_L	-2.84	0.0077	0.0257	u	0.0050
│ │ │ ├── THALAMUS-VIM_L	-2.16	0.0380	0.0633	u	0.0050
│ │ │ ├── THALAMUS-VLA_L	-2.61	0.0136	0.0340	u	0.0050
│ │ │ ├── THALAMUS-VM_L	-2.84	0.0077	0.0257	u	0.0050
│ │ │ └── THALAMUS-VPMPC_L	-2.23	0.0332	0.0633	u	0.0050
│ │ └── VENTRAL_THALAMUS_L	-2.29	0.0050	0.0135	**	0.0375
│ │ └── THALAMUS-ZI_L	-3.28	0.0025	0.0050	**	0.0250
│ ├── MESENCEPHALON_L	-1.93	0.0357	0.0476	*	0.0500
│ │ └── MIDBRAIN_TEGMENTUM_L	-2.48	0.0357	0.0357	*	0.0375
│ │ ├── NUCLEUS_RUBER_PARVOCELL_PART_L	-2.49	0.0180	0.0357	*	0.0375
│ │ │ └── MIDBRAIN-NRP_L	-2.49	0.0180	0.0180	*	0.0250
│ │ └── SUBSTANTIA_NIGRA_L	-2.53	0.0238	0.0357	*	0.0375
│ │ ├── MIDBRAIN-SNC_L	-2.36	0.0246	0.0246	*	0.0250
│ │ └── MIDBRAIN-SNR_L	-2.67	0.0119	0.0238	*	0.0250
│ ├── METENCEPHALON_L	-2.26	0.0099	0.0198	**	0.0500
│ │ ├── CEREBELLUM_L	-2.11	0.0099	0.0099	**	0.0375
│ │ │ ├── CEREBELLAR_CORTEX_L	-2.01	0.0348	0.0522	u	0.0375
│ │ │ │ ├── CEREBELLUM_ANT_LOBE_L	-2.97	0.0116	0.0348	u	0.0125
│ │ │ │ │ ├── Cerebellum_3_L	-2.68	0.0116	0.0116	u	0.0000
│ │ │ │ │ └── Cerebellum_4_5_L	-2.95	0.0059	0.0116	u	0.0000
│ │ │ │ ├── CEREBELLUM_POST_LOBE_L	-1.67	0.1330	0.1330		0.0125
│ │ │ │ │ ├── Cerebellum_6_L	-2.05	0.0489	0.1410	u	0.0000
│ │ │ │ │ └── Cerebellum_9_L	-2.47	0.0190	0.1330	u	0.0000
│ │ │ │ └── CEREBELLUM_VERMIS	-2.69	0.0946	0.1330		0.0125
│ │ │ │ ├── Vermis_3	-2.14	0.0403	0.0946	u	0.0000
│ │ │ │ ├── Vermis_4_5	-2.20	0.0348	0.0946	u	0.0000
│ │ │ │ ├── Vermis_6	-2.55	0.0156	0.0946	u	0.0000
│ │ │ │ └── Vermis_8	-2.06	0.0473	0.0946	u	0.0000
│ │ │ ├── CEREBELLAR_NUCLEI_L	-2.37	0.0578	0.0578		0.0375
│ │ │ │ └── DENTATE_NUCLEUS_L	-2.19	0.0454	0.0578	u	0.0125
│ │ │ │ ├── CEREBELLUM-NDENTD_L	-2.06	0.0476	0.0476	u	0.0000
│ │ │ │ └── CEREBELLUM-NDENTV_L	-2.39	0.0227	0.0454	u	0.0000
│ │ │ └── CEREBELLAR_WM_L	-3.18	0.0033	0.0099	**	0.0375
│ │ └── PONS_L	-3.01	0.0062	0.0099	**	0.0375
│ │ ├── PONTINE_TEGMENTUM_L	-2.31	0.0274	0.0274	*	0.0375
│ │ └── VENTRAL_PONS_L	-3.20	0.0031	0.0062	**	0.0375
│ └── TELENCEPHALON_L	-0.90	0.2080	0.2080		0.0500
│ ├── CEREBRAL_CORTEX_L	-0.72	0.2080	0.2080		0.0375
│ │ ├── FRONTAL_LOBE_L	-1.70	0.3680	0.3680		0.0000
│ │ │ ├── DORSAL_PRECENTRAL_GYRUS_L	-2.42	0.0186	0.3680	u	0.0000
│ │ │ │ └── AREA-6D1_L	-2.77	0.0093	0.0186	u	0.0000
│ │ │ ├── LATERAL_ORBITOFRONTAL_CORTEX_L	-1.10	0.1800	0.5390		0.0000
│ │ │ │ └── AREA-FO6_L	-2.09	0.0449	0.1800	u	0.0000
│ │ │ └── MEDIAL_ORBITOFRONTAL_CORTEX_L	-1.08	0.0668	0.3680		0.0000
│ │ │ ├── AREA-FO1_L	-2.33	0.0262	0.0668	u	0.0000
│ │ │ └── AREA-FO2_L	-2.09	0.0445	0.0668	u	0.0000
│ │ ├── INSULA_L	-2.32	0.0468	0.2080	u	0.0000
│ │ │ ├── DYSGRANULAR_INSULA_L	-2.02	0.1470	0.2210		0.0000
│ │ │ │ ├── AREA-ID4_L	-2.52	0.0168	0.1470	u	0.0000
│ │ │ │ └── AREA-ID5_L	-2.28	0.0295	0.1470	u	0.0000
│ │ │ └── GRANULAR_INSULA_L	-2.89	0.0156	0.0468	u	0.0000
│ │ │ ├── AREA-IG1_L	-2.72	0.0104	0.0156	u	0.0000

REGION	t_uncorr	p_simes	p_fdr	sign	q_set
│ │ │ ├── AREA-IG2_L	-2.44	0.0206	0.0206	u	0.0000
│ │ │ └── AREA-IG3_L	-2.75	0.0097	0.0156	u	0.0000
│ │ ├── LIMBIC_LOBE_L	0.95	0.1100	0.2210		0.0000
│ │ │ ├── HIPPOCAMPAL_FORMATION_L	1.09	0.0552	0.1100		0.0000
│ │ │ │ └── AREA-EC_L	2.89	0.0069	0.0552	u	0.0000
│ │ │ └── PIRIFORM_CORTEX_L	2.25	0.0384	0.1100	u	0.0000
│ │ │ └── AREA-PIRT_L	2.66	0.0192	0.0384	u	0.0000
│ │ │ ├── AREA-PIRT.TIT_L	2.47	0.0192	0.0192	u	0.0000
│ │ │ └── AREA-PIRT.TU_L	2.62	0.0135	0.0192	u	0.0000
│ │ ├── OCCIPITAL_LOBE_L	-0.68	0.3590	0.3680		0.0000
│ │ │ └── LATERAL_OCCIPITAL_CORTEX_L	-2.05	0.0897	0.3590		0.0000
│ │ │ └── AREA-HOC4LP_L	-2.27	0.0299	0.0897	u	0.0000
│ │ ├── PARIETAL_LOBE_L	-0.57	0.3510	0.3680		0.0000
│ │ │ ├── INFERIOR_PARIETAL_LOBULE_L	-0.13	0.1330	0.3510		0.0000
│ │ │ │ └── AREA-PFCM_L	-2.47	0.0190	0.1330	u	0.0000
│ │ │ ├── INTRAPARIETAL_SULCUS_L	-2.00	0.1760	0.3510		0.0000
│ │ │ │ ├── AREA-HIP3_L	-2.10	0.0439	0.1760	u	0.0000
│ │ │ │ └── AREA-HIP4_L	-2.32	0.0272	0.1760	u	0.0000
│ │ │ └── PARIETAL_OPERCULUM_L	-0.05	0.0602	0.3510		0.0000
│ │ │ ├── AREA-OP2_L	-2.27	0.0301	0.0602	u	0.0000
│ │ │ └── AREA-OP4_L	2.54	0.0163	0.0602	u	0.0000
│ │ └── TEMPORAL_LOBE_L	1.31	0.0693	0.2080		0.0000
│ │ ├── COLLATERAL_SULCUS_L	2.92	0.0063	0.0693	u	0.0000
│ │ │ └── AREA-COS1_L	2.92	0.0063	0.0063	u	0.0000
│ │ ├── FUSIFORM_GYRUS_L	-0.37	0.1380	0.2280		0.0000
│ │ │ └── AREA-FG5_L	2.31	0.0275	0.1380	u	0.0000
│ │ ├── GAPMAP-TEMPORAL-TO-PARIETAL_L	2.05	0.0491	0.2280	u	0.0000
│ │ ├── HESCHL_GYRUS_L	-1.95	0.0738	0.2280		0.0000
│ │ │ └── AREA-TE-1.1_L	-2.36	0.0246	0.0738	u	0.0000
│ │ └── SUPERIOR_TEMPORAL_GYRUS_L	-0.93	0.0981	0.2280		0.0000
│ │ └── AREA-TE-2.2_L	-2.23	0.0327	0.0981	u	0.0000
│ └── CEREBRAL_NUCLEI_L	-1.20	0.1670	0.2080		0.0375
│ └── BASAL_GANGLIA_L	-1.09	0.0558	0.1670		0.0000
│ ├── Pallidum_L	-2.77	0.0093	0.0558	u	0.0000
│ └── VENTRAL_PALLIDUM_L	-2.41	0.0217	0.0651	u	0.0000
│ └── VENTRALPALLIDUM-VP_L	-2.41	0.0217	0.0217	u	0.0000
└── BRAIN_R	-1.99	0.0240	0.0240	*	0.0500
├── DIENCEPHALON_R	-1.28	0.0136	0.0272	*	0.0500
│ ├── SUBTHALAMUS_R	-2.90	0.0068	0.0136	**	0.0500
│ │ └── SUBTHALAMUS-STN_R	-2.90	0.0068	0.0068	**	0.0250
│ └── THALAMUS_R	-1.27	0.0660	0.0660		0.0500
│ ├── DORSAL_THALAMUS_R	-1.25	0.0450	0.0675	u	0.0250
│ │ ├── ANTERIOR_GROUP_R	-3.04	0.0117	0.0450	u	0.0000
│ │ │ ├── THALAMUS-AM_R	-3.11	0.0039	0.0117	u	0.0000
│ │ │ └── THALAMUS-AV_R	-2.84	0.0078	0.0117	u	0.0000
│ │ ├── MEDIAL_GROUP_R	-0.98	0.0180	0.0450	u	0.0000
│ │ │ └── THALAMUS-PV_R	-2.94	0.0060	0.0180	u	0.0000
│ │ └── VENTRAL_GROUP_R	-1.90	0.0395	0.0658	u	0.0000
│ │ ├── THALAMUS-VAMC_R	-2.83	0.0079	0.0395	u	0.0000
│ │ ├── THALAMUS-VA_R	-3.10	0.0040	0.0395	u	0.0000
│ │ ├── THALAMUS-VLA_R	-2.50	0.0179	0.0447	u	0.0000
│ │ └── THALAMUS-VM_R	-2.51	0.0173	0.0447	u	0.0000
│ └── VENTRAL_THALAMUS_R	-1.34	0.0220	0.0660	u	0.0250
│ └── THALAMUS-ZI_R	-2.70	0.0110	0.0220	u	0.0000
├── MESENCEPHALON_R	-2.14	0.0303	0.0404	*	0.0500
│ └── MIDBRAIN_TEGMENTUM_R	-2.64	0.0303	0.0303	*	0.0500
│ ├── NUCLEUS_RUBER_PARVOCELL_PART_R	-2.45	0.0202	0.0303	*	0.0500
│ │ └── MIDBRAIN-NRP_R	-2.45	0.0202	0.0202	*	0.0333
│ └── SUBSTANTIA_NIGRA_R	-2.67	0.0144	0.0303	*	0.0500
│ ├── MIDBRAIN-SNC_R	-2.43	0.0207	0.0207	*	0.0333
│ └── MIDBRAIN-SNR_R	-2.87	0.0072	0.0144	**	0.0333
├── METENCEPHALON_R	-2.80	0.0060	0.0240	**	0.0500
│ ├── CEREBELLUM_R	-2.63	0.0039	0.0060	**	0.0500
│ │ ├── CEREBELLAR_CORTEX_R	-2.46	0.0792	0.0792		0.0500
│ │ │ ├── CEREBELLUM_ANT_LOBE_R	-2.37	0.0396	0.0792	u	0.0167
│ │ │ │ └── Cerebellum_4_5_R	-2.45	0.0198	0.0396	u	0.0000
│ │ │ └── CEREBELLUM_POST_LOBE_R	-2.30	0.0805	0.0805		0.0167
│ │ │ ├── Cerebellum_6_R	-2.66	0.0121	0.0805	u	0.0000
│ │ │ ├── Cerebellum_9_R	-2.29	0.0288	0.0805	u	0.0000
│ │ │ └── Cerebellum_Crus1_R	-2.21	0.0345	0.0805	u	0.0000
│ │ ├── CEREBELLAR_NUCLEI_R	-2.64	0.0437	0.0655	u	0.0500
│ │ │ ├── CEREBELLUM-NINTERP_R	-2.10	0.0437	0.0437	u	0.0167
│ │ │ ├── DENTATE_NUCLEUS_R	-2.45	0.0292	0.0437	u	0.0167
│ │ │ │ ├── CEREBELLUM-NDENTD_R	-2.24	0.0322	0.0322	u	0.0000
│ │ │ │ └── CEREBELLUM-NDENTV_R	-2.58	0.0146	0.0292	u	0.0000

REGION	t_uncorr	p_simes	p_fdr	sign	q_set
│ │ │ └── FASTIGIAL_NUCLEUS_R	-2.10	0.0433	0.0437	u	0.0167
│ │ │ └── CEREBELLUM-NFAST_R	-2.10	0.0433	0.0433	u	0.0000
│ │ └── CEREBELLAR_WM_R	-3.52	0.0013	0.0039	**	0.0500
│ └── PONS_R	-3.08	0.0060	0.0060	**	0.0500
│ ├── PONTINE_TEGMENTUM_R	-2.51	0.0175	0.0175	*	0.0500
│ └── VENTRAL_PONS_R	-3.21	0.0030	0.0060	**	0.0500
└── TELENCEPHALON_R	-1.14	0.0450	0.0450	*	0.0500
├── CEREBRAL_CORTEX_R	-0.81	0.7590	0.7590		0.0500
│ ├── FRONTAL_LOBE_R	-1.73	0.3890	0.7590		0.0250
│ │ ├── FRONTAL_OPERCULUM_R	-1.25	0.2630	0.8190		0.0000
│ │ │ └── AREA-OP5_R	-2.10	0.0438	0.2630	u	0.0000
│ │ ├── MEDIAL_ORBITOFRONTAL_CORTEX_R	-1.86	0.0201	0.3890	u	0.0000
│ │ │ ├── AREA-FO1_R	-2.90	0.0067	0.0201	u	0.0000
│ │ │ └── AREA-FO2_R	-2.05	0.0483	0.0725	u	0.0000
│ │ └── SUPERIOR_FRONTAL_SULCUS_R	-2.90	0.0522	0.3890		0.0000
│ │ ├── AREA-SFG3_R	-2.35	0.0253	0.0522	u	0.0000
│ │ └── AREA-SFS1_R	-2.33	0.0261	0.0522	u	0.0000
│ ├── INSULA_R	-1.88	0.2880	0.7590		0.0250
│ │ └── DYSGRANULAR_INSULA_R	-1.77	0.0960	0.2880		0.0000
│ │ └── AREA-ID4_R	-2.76	0.0096	0.0960	u	0.0000
│ ├── OCCIPITAL_LOBE_R	-1.24	0.5060	0.7590		0.0250
│ │ └── LATERAL_OCCIPITAL_CORTEX_R	-1.56	0.1500	0.5060		0.0000
│ │ └── AREA-HOC5_R	-2.04	0.0499	0.1500	u	0.0000
│ └── TEMPORAL_LOBE_R	1.37	0.3870	0.7590		0.0250
│ ├── COLLATERAL_SULCUS_R	2.20	0.0352	0.3870	u	0.0000
│ │ └── AREA-COS1_R	2.20	0.0352	0.0352	u	0.0000
│ └── FUSIFORM_GYRUS_R	0.72	0.1260	0.4140		0.0000
│ └── AREA-FG5_R	2.35	0.0252	0.1260	u	0.0000
└── CEREBRAL_NUCLEI_R	-2.13	0.0225	0.0450	*	0.0500
├── AMYGDALA_R	-0.45	0.5020	0.5020		0.0250
│ ├── AMYGDALA-MF_R	-1.90	0.0864	0.5020		0.0083
│ │ └── AMYGDALA-MF.LM_R	-2.11	0.0432	0.0864	u	0.0000
│ └── AMYGDALA-SF_R	-0.50	0.1430	0.5020		0.0083
│ └── AMYGDALA-SF.AHI_R	-2.06	0.0478	0.1430	u	0.0000
├── BASAL_FOREBRAIN_R	-1.85	0.0237	0.0356	u	0.0250
│ └── SUBLENTICULAR_BASAL_FOREBRAIN_R	-2.83	0.0079	0.0237	u	0.0083
│ └── BASALFOREBRAIN-CH-4_R	-2.83	0.0079	0.0079	u	0.0000
└── BASAL_GANGLIA_R	-2.03	0.0075	0.0225	**	0.0250
├── N_Acc_R	-2.22	0.0333	0.0500	u	0.0083
├── Pallidum_R	-3.28	0.0025	0.0075	**	0.0083
├── VENTRAL_PALLIDUM_R	-3.34	0.0022	0.0075	**	0.0083
│ └── VENTRALPALLIDUM-VP_R	-3.34	0.0022	0.0022	**	0.0028
└── VENTRAL_STRIATUM_R	-2.95	0.0082	0.0164	u	0.0083
├── VENTRALSTRIATUM-ACBL_R	-3.19	0.0032	0.0082	u	0.0028
├── VENTRALSTRIATUM-FUCD_R	-2.22	0.0339	0.0452	u	0.0028
└── VENTRALSTRIATUM-FUP_R	-3.10	0.0041	0.0082	u	0.0028

Table S2: Associations between PCL-R dimension 2 and regions of the hierarchical brain atlas. p-values of higher-level regions where calculated using the Simes method (see above and main text).

Table S3: Associations between PCL-R total and regions of hierarchical brain atlas

REGION	t_uncorr	p_simes	p_fdr	sign	q_set
BRAIN	-0.84	0.6000	0.6000		0.0500
├── BRAIN_L	-0.72	0.6000	0.6000		0.0500
│ ├── METENCEPHALON_L	-2.18	0.1500	0.6000		0.0000
│ │ └── CEREBELLUM_L	-2.17	0.1110	0.1500		0.0000
│ │ ├── CEREBELLAR_CORTEX_L	-2.16	0.1820	0.2230		0.0000
│ │ │ ├── CEREBELLUM_POST_LOBE_L	-2.06	0.1410	0.1820		0.0000
│ │ │ │ └── Cerebellum_8_L	-2.23	0.0329	0.1410	u	0.0000
│ │ │ └── CEREBELLUM_VERMIS	-1.98	0.1820	0.1820		0.0000
│ │ │ └── Vermis_8	-2.39	0.0228	0.1820	u	0.0000
│ │ └── CEREBELLAR_WM_L	-2.17	0.0369	0.1110	u	0.0000
│ └── TELENCEPHALON_L	0.02	0.6010	0.8020		0.0000
│ └── CEREBRAL_CORTEX_L	-0.02	0.3010	0.6010		0.0000
│ ├── FRONTAL_LOBE_L	-0.78	0.1700	0.3010		0.0000
│ │ └── MEDIAL_ORBITOFRONTAL_CORTEX_L	-1.55	0.0081	0.1700	u	0.0000
│ │ ├── AREA-FO1_L	-2.89	0.0067	0.0100	u	0.0000
│ │ └── AREA-FO2_L	-3.25	0.0027	0.0081	u	0.0000
│ ├── LIMBIC_LOBE_L	1.57	0.0544	0.3010		0.0000
│ │ ├── HIPPOCAMPAL_FORMATION_L	2.28	0.0272	0.0544	u	0.0000
│ │ │ └── AREA-EC_L	3.16	0.0034	0.0272	u	0.0000
│ │ └── PIRIFORM_CORTEX_L	2.40	0.0152	0.0544	u	0.0000
│ │ └── AREA-PIRT_L	2.71	0.0076	0.0152	u	0.0000
│ │ ├── AREA-PIRT.TIT_L	2.37	0.0240	0.0240	u	0.0000
│ │ └── AREA-PIRT.TU_L	3.11	0.0038	0.0076	u	0.0000
│ ├── PARIETAL_LOBE_L	-0.14	0.2000	0.3010		0.0000
│ │ ├── INFERIOR_PARIETAL_LOBULE_L	-0.13	0.2450	0.5440		0.0000
│ │ │ └── AREA-PFCM_L	-2.20	0.0350	0.2450	u	0.0000
│ │ └── PARIETO-OCCIPITAL_SULCUS_L	2.22	0.0334	0.2000	u	0.0000
│ │ └── AREA-HPO1_L	2.22	0.0334	0.0334	u	0.0000
│ └── TEMPORAL_LOBE_L	1.67	0.1280	0.3010		0.0000
│ ├── COLLATERAL_SULCUS_L	2.23	0.0324	0.1280	u	0.0000
│ │ └── AREA-COS1_L	2.23	0.0324	0.0324	u	0.0000
│ ├── GAPMAP-TEMPORAL-TO-PARIETAL_L	2.45	0.0196	0.1280	u	0.0000
│ └── SUPERIOR_TEMPORAL_GYRUS_L	-1.19	0.0348	0.1280	u	0.0000
│ └── AREA-TE-2.2_L	-2.67	0.0116	0.0348	u	0.0000
└── BRAIN_R	-0.95	0.5290	0.6000		0.0500
├── METENCEPHALON_R	-2.23	0.1320	0.5290		0.0000
│ └── CEREBELLUM_R	-2.19	0.1320	0.1320		0.0000
│ └── CEREBELLAR_CORTEX_R	-2.16	0.0882	0.1320		0.0000
│ └── CEREBELLUM_POST_LOBE_R	-2.15	0.0441	0.0882	u	0.0000
│ ├── Cerebellum_6_R	-2.64	0.0126	0.0441	u	0.0000
│ └── Cerebellum_Crus1_R	-2.87	0.0070	0.0441	u	0.0000
└── TELENCEPHALON_R	-0.30	0.3020	0.6050		0.0000
├── CEREBRAL_CORTEX_R	-0.28	0.1510	0.3020		0.0000
│ ├── FRONTAL_LOBE_R	-1.45	0.0252	0.1510	u	0.0000
│ │ ├── FRONTAL_OPERCULUM_R	-1.32	0.1210	0.6130		0.0000
│ │ │ └── AREA-OP5_R	-2.44	0.0202	0.1210	u	0.0000
│ │ ├── MEDIAL_ORBITOFRONTAL_CORTEX_R	-2.31	0.0012	0.0252	u	0.0000
│ │ │ ├── AREA-FO1_R	-3.92	0.0004	0.0012	u	0.0000
│ │ │ └── AREA-FO2_R	-3.24	0.0028	0.0042	u	0.0000
│ │ ├── POSTERIOR_SFG_R	-2.09	0.0447	0.3130	u	0.0000
│ │ │ └── AREA-6MA_R	-2.09	0.0447	0.0447	u	0.0000
│ │ ├── SUPERIOR_FRONTAL_GYRUS_R	-2.53	0.0124	0.1300	u	0.0000
│ │ │ └── AREA-8D2_R	-3.19	0.0031	0.0124	u	0.0000
│ │ └── SUPERIOR_FRONTAL_SULCUS_R	-2.74	0.1460	0.6130		0.0000
│ │ └── AREA-SFG3_R	-2.18	0.0365	0.1460	u	0.0000
│ ├── INSULA_R	-1.27	0.4380	0.8780		0.0000
│ │ └── DYSGRANULAR_INSULA_R	-1.34	0.1460	0.4380		0.0000
│ │ └── AREA-ID4_R	-2.58	0.0146	0.1460	u	0.0000
│ └── LIMBIC_LOBE_R	0.61	0.6050	0.8780		0.0000
│ └── HIPPOCAMPAL_FORMATION_R	2.07	0.2360	0.6050		0.0000
│ └── HIPPOCAMPUS-CA1_R	2.28	0.0295	0.2360	u	0.0000
└── CEREBRAL_NUCLEI_R	-0.60	0.7610	0.7610		0.0000
└── BASAL_GANGLIA_R	-0.60	0.4840	0.7610		0.0000
└── VENTRAL_STRIATUM_R	-1.51	0.1640	0.4910		0.0000
└── VENTRALSTRIATUM-ACBL_R	-2.13	0.0409	0.1640	u	0.0000

Table S3: Associations between PCL-R total score and regions of the hierarchical brain atlas. p-values of higher-level regions where calculated using the Simes method (see above and main text).

Figure S4: Associations between PCL-R total and brain regions

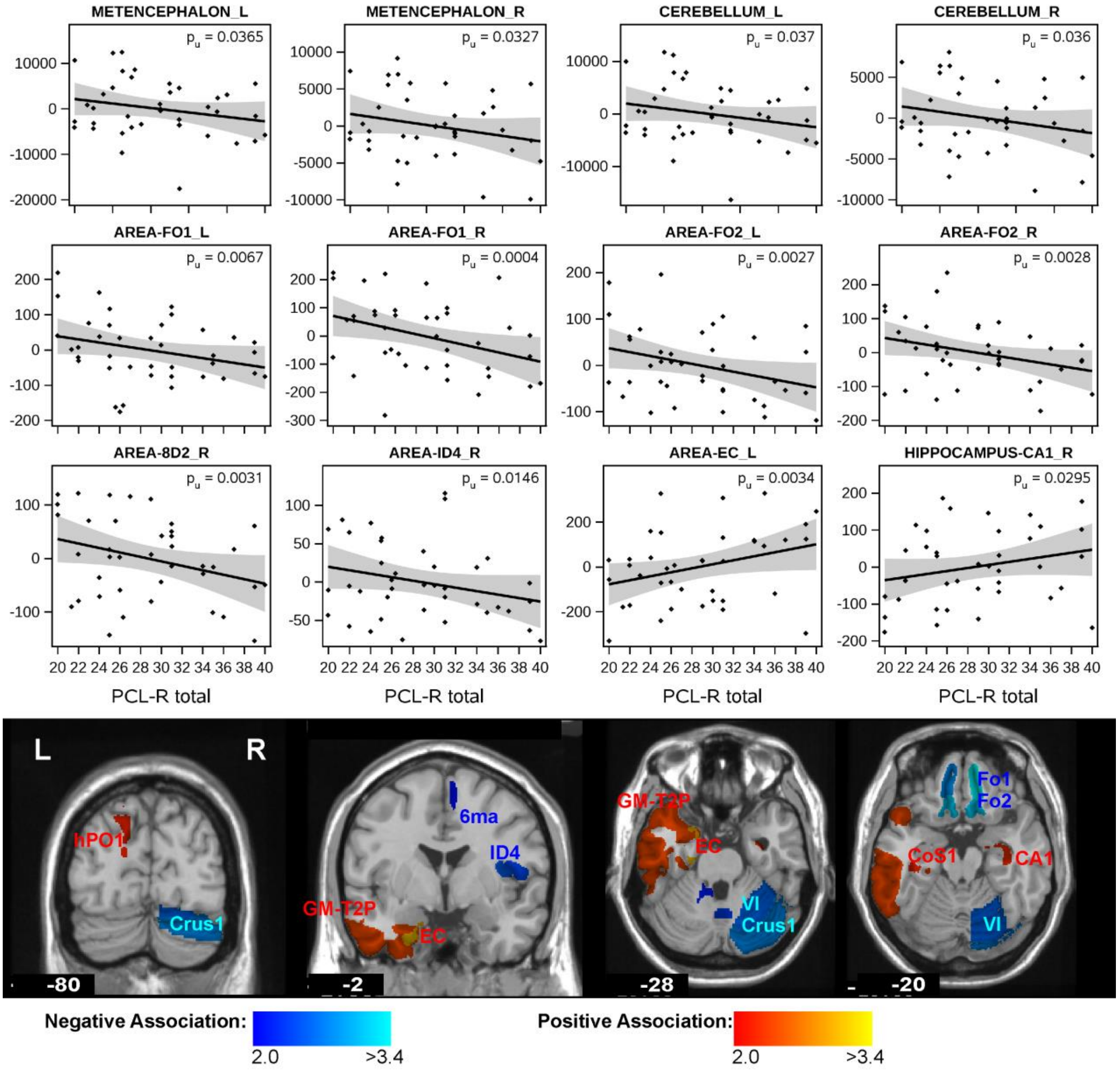

Figure S4: Associations of PCL-R total with volumes of brain regions in psychopathic subjects with PCL-R total ≥ 20. Volume data were adjusted for study (acquisition site), age and intra-cranial volume. The scatter-plots show the individual data points, the regression line with its confidence limits and the p-value (uncorr.). The bottom row shows the brain regions, where an association with $p_{uncorr} < 0.05$ had been found, with colors corresponding to the statistical score (student's t). Blue-cyan: Negative association, red-yellow: positive association.

Table S5: Group differences between psychopathic subjects and controls in regions of the hierarchical atlas

REGION	t_uncorr	p_uncorr	p_fdr	sign	q_set
BRAIN	-2.96	0.0042	0.0042	**	0.0500
├── BRAIN_L	-2.95	0.0043	0.0061	**	0.0500
│ ├── METENCEPHALON_L	-1.87	0.0662	0.1320		0.0500
│ │ └── CEREBELLUM_L	-1.96	0.0542	0.1080		0.0000
│ │ ├── CEREBELLAR_CORTEX_L	-1.96	0.0537	0.0805		0.0000
│ │ │ ├── CEREBELLUM_POST_LOBE_L	-2.20	0.0310	0.0930	u	0.0000
│ │ │ │ └── Cerebellum_6_L	-2.20	0.0310	0.0933	u	0.0000
│ │ │ └── CEREBELLUM_VERMIS	-0.38	0.7040	0.8320		0.0000
│ │ │ └── Vermis_10	-2.43	0.0176	0.1410	u	0.0000
│ │ └── CEREBELLAR_NUCLEI_L	-2.07	0.0423	0.0805	u	0.0000
│ │ └── DENTATE_NUCLEUS_L	-1.90	0.0612	0.1840		0.0000
│ │ └── CEREBELLUM-NDENTD_L	-2.05	0.0436	0.0872	u	0.0000
│ └── TELENCEPHALON_L	-2.46	0.0162	0.0648	u	0.0500
│ ├── CEREBRAL_CORTEX_L	-2.83	0.0061	0.0122	u	0.0000
│ │ ├── FRONTAL_LOBE_L	-2.61	0.0111	0.0666	u	0.0000
│ │ │ ├── GAPMAP-FRONTAL-TO-OCCIPITAL_L	-2.51	0.0142	0.2980	u	0.0000
│ │ │ ├── MIDDLE_FRONTAL_GYRUS_L	-1.78	0.0789	0.3260		0.0000
│ │ │ │ └── AREA-8V2_L	-2.15	0.0353	0.1410	u	0.0000
│ │ │ ├── SUPERIOR_FRONTAL_GYRUS_L	-1.88	0.0636	0.3260		0.0000
│ │ │ │ ├── AREA-SFG2_L	-2.20	0.0309	0.0724	u	0.0000
│ │ │ │ └── AREA-SFG4_L	-2.13	0.0362	0.0724	u	0.0000
│ │ │ └── SUPERIOR_FRONTAL_SULCUS_L	-1.62	0.1090	0.3260		0.0000
│ │ │ └── AREA-6D3_L	-2.31	0.0236	0.0944	u	0.0000
│ │ ├── LIMBIC_LOBE_L	-1.95	0.0548	0.1110		0.0000
│ │ │ └── HIPPOCAMPAL_FORMATION_L	-1.78	0.0796	0.3180		0.0000
│ │ │ └── HIPPOCAMPUS-SUBC_L	-1.97	0.0531	0.1890		0.0000
│ │ │ └── HIPPOCAMPUS-SUBC.PRES_L	-2.10	0.0390	0.1160	u	0.0000
│ │ ├── OCCIPITAL_LOBE_L	-0.96	0.3400	0.3400		0.0000
│ │ │ └── DORSAL_OCCIPITAL_CORTEX_L	-2.06	0.0431	0.1720	u	0.0000
│ │ │ ├── AREA-HOC4D_L	-2.44	0.0173	0.0516	u	0.0000
│ │ │ └── AREA-HOC6_L	-2.16	0.0344	0.0516	u	0.0000
│ │ ├── PARIETAL_LOBE_L	-1.56	0.1220	0.1840		0.0000
│ │ │ ├── INFERIOR_PARIETAL_LOBULE_L	-0.95	0.3460	0.4800		0.0000
│ │ │ │ └── AREA-PGP_L	-2.16	0.0339	0.2370	u	0.0000
│ │ │ ├── INTRAPARIETAL_SULCUS_L	-2.79	0.0067	0.0402	u	0.0000
│ │ │ │ ├── AREA-HIP1_L	-3.89	0.0002	0.0016	u	0.0000
│ │ │ │ ├── AREA-HIP2_L	-2.70	0.0087	0.0232	u	0.0000
│ │ │ │ └── AREA-HIP3_L	-2.75	0.0075	0.0232	u	0.0000
│ │ │ └── PARIETAL_OPERCULUM_L	1.06	0.2930	0.4800		0.0000
│ │ │ └── AREA-OP1_L	2.44	0.0174	0.0696	u	0.0000
│ │ └── TEMPORAL_LOBE_L	-1.95	0.0555	0.1110		0.0000
│ │ ├── GAPMAP-FRONTAL-TO-TEMPORAL-II_L	-2.04	0.0450	0.1240	u	0.0000
│ │ ├── OCCIPITO-TEMPORAL_SULCUS_L	2.57	0.0122	0.1240	u	0.0000
│ │ │ └── AREA-OTS1_L	2.57	0.0122	0.0122	u	0.0000
│ │ ├── PARAHIPPOCAMPAL_GYRUS_L	-2.31	0.0235	0.1240	u	0.0000
│ │ │ ├── AREA-PH2_L	-2.00	0.0494	0.0741	u	0.0000
│ │ │ └── AREA-PH3_L	-2.49	0.0152	0.0456	u	0.0000
│ │ └── SUPERIOR_TEMPORAL_SULCUS_L	-2.16	0.0338	0.1240	u	0.0000
│ │ └── AREA-STS2_L	-2.06	0.0435	0.0809	u	0.0000
│ └── CEREBRAL_NUCLEI_L	-0.53	0.6000	0.6000		0.0000
│ └── AMYGDALA_L	-1.53	0.1300	0.3890		0.0000
│ ├── AMYGDALA-CM_L	-1.52	0.1340	0.2350		0.0000
│ │ └── AMYGDALA-CM.ME_L	-2.06	0.0426	0.1280	u	0.0000
│ ├── AMYGDALA-LB_L	-1.29	0.2010	0.2820		0.0000
│ │ └── AMYGDALA-LB.BM_L	-2.29	0.0251	0.1000	u	0.0000
│ └── AMYGDALA-MF_L	-2.07	0.0418	0.2350	u	0.0000
│ └── AMYGDALA-MF.ICM_L	-2.22	0.0296	0.0592	u	0.0000
└── BRAIN_R	-2.83	0.0061	0.0061	**	0.0500
├── DIENCEPHALON_R	-0.58	0.5630	0.5630		0.0500
│ └── THALAMUS_R	-0.59	0.5540	0.9080		0.0125
│ └── METATHALAMUS_R	-1.68	0.0969	0.2910		0.0000
│ └── LATERAL_GENICULATE_BODY_R	-2.14	0.0356	0.0712	u	0.0000
│ └── METATHALAMUS-CGL_R	-2.14	0.0356	0.0356	u	0.0000
└── TELENCEPHALON_R	-2.65	0.0098	0.0392	**	0.0500
├── CEREBRAL_CORTEX_R	-3.10	0.0028	0.0056	**	0.0125
│ ├── FRONTAL_LOBE_R	-1.75	0.0843	0.1010		0.0063
│ │ ├── DORSAL_PRECENTRAL_GYRUS_R	-1.89	0.0626	0.3290		0.0000
│ │ │ └── AREA-6D2_R	-2.00	0.0493	0.0986	u	0.0000

REGION	t_uncorr	p_uncorr	p_fdr	sign	q_set
│ │ ├── GAPMAP-FRONTAL-TO-OCCIPITAL_R	-2.89	0.0051	0.0672	u	0.0000
│ │ ├── INFERIOR_FRONTAL_SULCUS_R	-1.47	0.1460	0.4700		0.0000
│ │ │ └── AREA-IFS1_R	-2.03	0.0458	0.2160	u	0.0000
│ │ ├── MESIAL_PRECENTRAL_GYRUS_R	-2.81	0.0064	0.0672	u	0.0000
│ │ │ └── AREA-6MP_R	-2.81	0.0064	0.0064	u	0.0000
│ │ ├── MIDDLE_FRONTAL_GYRUS_R	-1.16	0.2490	0.5820		0.0000
│ │ │ └── AREA-MFG4_R	-2.32	0.0230	0.0920	u	0.0000
│ │ ├── POSTERIOR_SFG_R	-2.58	0.0121	0.0847	u	0.0000
│ │ │ └── AREA-6MA_R	-2.58	0.0121	0.0121	u	0.0000
│ │ └── SUPERIOR_FRONTAL_SULCUS_R	-1.43	0.1570	0.4700		0.0000
│ │ └── AREA-6D3_R	-2.04	0.0454	0.1820	u	0.0000
│ ├── INSULA_R	-2.54	0.0132	0.0396	u	0.0063
│ │ └── DYSGRANULAR_INSULA_R	-2.46	0.0161	0.0483	u	0.0000
│ │ ├── AREA-ID4_R	-2.17	0.0331	0.1650	u	0.0000
│ │ └── AREA-ID9_R	-2.20	0.0309	0.1650	u	0.0000
│ ├── LIMBIC_LOBE_R	-2.91	0.0048	0.0288	u	0.0063
│ │ ├── FRONTAL_CINGULATE_GYRUS_R	-1.33	0.1870	0.2490		0.0000
│ │ │ └── AREA-33_R	-2.14	0.0355	0.2260	u	0.0000
│ │ ├── HIPPOCAMPAL_FORMATION_R	-3.17	0.0022	0.0088	u	0.0000
│ │ │ ├── HIPPOCAMPUS-CA1_R	-2.17	0.0332	0.1330	u	0.0000
│ │ │ └── HIPPOCAMPUS-SUBC_R	-4.37	0.0000	0.0000	u	0.0000
│ │ │ ├── HIPPOCAMPUS-SUBC.PAS_R	-2.60	0.0112	0.0112	u	0.0000
│ │ │ ├── HIPPOCAMPUS-SUBC.PRES_R	-3.37	0.0012	0.0016	u	0.0000
│ │ │ ├── HIPPOCAMPUS-SUBC.PROS_R	-4.09	0.0001	0.0002	u	0.0000
│ │ │ └── HIPPOCAMPUS-SUBC.SUB_R	-4.14	0.0001	0.0002	u	0.0000
│ │ └── PIRIFORM_CORTEX_R	-1.60	0.1130	0.2260		0.0000
│ │ └── AREA-PIRTB_R	-2.28	0.0255	0.0510	u	0.0000
│ │ ├── AREA-PIRTB.TBD_R	-2.26	0.0272	0.0369	u	0.0000
│ │ └── AREA-PIRTB.TBV_R	-2.13	0.0369	0.0369	u	0.0000
│ ├── PARIETAL_LOBE_R	-2.24	0.0280	0.0466	u	0.0063
│ │ ├── INFERIOR_PARIETAL_LOBULE_R	-1.54	0.1270	0.2540		0.0000
│ │ │ └── AREA-PFCM_R	-2.12	0.0375	0.2630	u	0.0000
│ │ ├── INTRAPARIETAL_SULCUS_R	-1.94	0.0564	0.2540		0.0000
│ │ │ └── AREA-HIP3_R	-2.07	0.0424	0.3390	u	0.0000
│ │ └── SUPERIOR_PARIETAL_LOBULE_R	-1.55	0.1260	0.2540		0.0000
│ │ └── AREA-7PC_R	-2.64	0.0103	0.0721	u	0.0000
│ └── TEMPORAL_LOBE_R	-2.20	0.0311	0.0466	u	0.0063
│ ├── GAPMAP-FRONTAL-TO-TEMPORAL-II_R	-2.74	0.0077	0.0847	u	0.0000
│ └── TEMPORO-PARIETAL_JUNCTION_R	-2.31	0.0240	0.1320	u	0.0000
│ └── AREA-TPJ_R	-2.31	0.0240	0.0240	u	0.0000
└── CEREBRAL_NUCLEI_R	-0.74	0.4600	0.4600		0.0125
└── AMYGDALA_R	-2.24	0.0281	0.0843	u	0.0063
├── AMYGDALA-CM_R	-1.99	0.0499	0.1160	u	0.0000
│ └── AMYGDALA-CM.CE_R	-2.08	0.0413	0.1240	u	0.0000
├── AMYGDALA-IF_R	-1.43	0.1570	0.1830		0.0000
│ └── AMYGDALA-IF.LD_R	-2.13	0.0370	0.1010	u	0.0000
├── AMYGDALA-LB_R	-1.86	0.0668	0.1170		0.0000
│ └── AMYGDALA-LB.BL_R	-2.25	0.0276	0.1100	u	0.0000
├── AMYGDALA-VTM_R	-2.39	0.0196	0.1160	u	0.0000
└── AMYGDALOSTRIATAL_TZ_R	-2.03	0.0459	0.1160	u	0.0000
└── AMYGDALA-ASTR_R	-2.03	0.0459	0.0459	u	0.0000

Table S5: Group differences between psychopathic subjects and controls in regions of the hierarchical brain atlas. p-values of all regions were calculated by direct evaluation of the statistical model with each region's volume data (see above and main text). The test statistic t_{uncorr} belongs to the comparison $V_{PS} – V_C$, thus a negative value indicates that the region volume was smaller in the brains of psychopathic subjects than in controls' brains.

Table S6: Hierarchical anatomical atlas

JB-3.1: Julich-Brain Atlas 3.1 (Amunts et al. 2020), https://www.ebrains.eu/tools/human-brain-atlas

AAL3: AAL3-Atlas (Tzourio-Mazoyer et al. 2002, Rolls et al. 2020)

```
BRAIN
└── DIENCEPHALON
    ├── SUBTHALAMUS
    │   └── SUBTHALAMUS-STN / JB-3.1
    └── THALAMUS
        ├── DORSAL_THALAMUS
        │   ├── ANTERIOR_GROUP
        │   │   ├── THALAMUS-AM / JB-3.1
        │   │   ├── THALAMUS-AV / JB-3.1
        │   │   └── THALAMUS-LD / JB-3.1
        │   ├── INTRALAMINAR_GROUP
        │   │   ├── THALAMUS-CL / JB-3.1
        │   │   ├── THALAMUS-CM / JB-3.1
        │   │   ├── THALAMUS-PF / JB-3.1
        │   │   └── THALAMUS-SPF / JB-3.1
        │   ├── MEDIAL_GROUP
        │   │   ├── THALAMUS-MD / JB-3.1
        │   │   ├── THALAMUS-MV / JB-3.1
        │   │   └── THALAMUS-PV / JB-3.1
        │   ├── POSTERIOR_GROUP
        │   │   ├── THALAMUS-LI / JB-3.1
        │   │   ├── THALAMUS-LP / JB-3.1
        │   │   ├── THALAMUS-PO / JB-3.1
        │   │   ├── THALAMUS-PUA / JB-3.1
        │   │   ├── THALAMUS-PUI / JB-3.1
        │   │   ├── THALAMUS-PUL / JB-3.1
        │   │   ├── THALAMUS-PUM / JB-3.1
        │   │   └── THALAMUS-SG / JB-3.1
        │   └── VENTRAL_GROUP
        │       ├── THALAMUS-VA / JB-3.1
        │       ├── THALAMUS-VAMC / JB-3.1
        │       ├── THALAMUS-VIM / JB-3.1
        │       ├── THALAMUS-VLA / JB-3.1
        │       ├── THALAMUS-VLP / JB-3.1
        │       ├── THALAMUS-VM / JB-3.1
        │       ├── THALAMUS-VPI / JB-3.1
        │       ├── THALAMUS-VPL / JB-3.1
        │       ├── THALAMUS-VPM / JB-3.1
        │       └── THALAMUS-VPMPC / JB-3.1
        ├── METATHALAMUS
        │   ├── LATERAL_GENICULATE_BODY
        │   │   └── METATHALAMUS-CGL / JB-3.1
        │   └── MEDIAL_GENICULATE_BODY
        │       └── METATHALAMUS-CGM / JB-3.1
        └── VENTRAL_THALAMUS
            ├── THALAMUS-RT / JB-3.1
            └── THALAMUS-ZI / JB-3.1
```

```
BRAIN
└── METENCEPHALON
    ├── CEREBELLUM
    │   ├── CEREBELLAR_CORTEX
    │   │   ├── CEREBELLUM_ANT_LOBE
    │   │   │   ├── Cerebellum_3 / AAL3
    │   │   │   └── Cerebellum_4_5 / AAL3
    │   │   ├── CEREBELLUM_POST_LOBE
    │   │   │   ├── Cerebellum_10 / AAL3
    │   │   │   ├── Cerebellum_6 / AAL3
    │   │   │   ├── Cerebellum_7b / AAL3
    │   │   │   ├── Cerebellum_8 / AAL3
    │   │   │   ├── Cerebellum_9 / AAL3
    │   │   │   ├── Cerebellum_Crus1 / AAL3
    │   │   │   └── Cerebellum_Crus2 / AAL3
    │   │   └── CEREBELLUM_VERMIS
    │   │       ├── Vermis_10 / AAL3
    │   │       ├── Vermis_1_2 / AAL3
    │   │       ├── Vermis_3 / AAL3
    │   │       ├── Vermis_4_5 / AAL3
    │   │       ├── Vermis_6 / AAL3
    │   │       ├── Vermis_7 / AAL3
    │   │       ├── Vermis_8 / AAL3
    │   │       └── Vermis_9 / AAL3
    │   ├── CEREBELLAR_NUCLEI
    │   │   ├── CEREBELLUM-NINTERP / JB-3.1
    │   │   ├── DENTATE_NUCLEUS
    │   │   │   ├── CEREBELLUM-NDENTD / JB-3.1
    │   │   │   └── CEREBELLUM-NDENTV / JB-3.1
    │   │   └── FASTIGIAL_NUCLEUS
    │   │       └── CEREBELLUM-NFAST / JB-3.1
    │   └── CEREBELLAR_WM
    └── PONS
        ├── PONTINE_TEGMENTUM
        └── VENTRAL_PONS
```

```
BRAIN
└── MESENCEPHALON
    └── MIDBRAIN_TEGMENTUM
        ├── NUCLEUS_RUBER_MAGNOCELL_PART
        │   └── MIDBRAIN-NRM / JB-3.1
        ├── NUCLEUS_RUBER_PARVOCELL_PART
        │   └── MIDBRAIN-NRP / JB-3.1
        └── SUBSTANTIA_NIGRA
            ├── MIDBRAIN-SNC / JB-3.1
            └── MIDBRAIN-SNR / JB-3.1
```

```
BRAIN
└── TELENCEPHALON
    └── CEREBRAL_CORTEX
        └── FRONTAL_LOBE
            ├── DORSAL_PRECENTRAL_GYRUS
            │   ├── AREA-6D1 / JB-3.1
            │   └── AREA-6D2 / JB-3.1
            ├── FRONTAL_OPERCULUM
            │   ├── AREA-OP10 / JB-3.1
            │   ├── AREA-OP5 / JB-3.1
            │   ├── AREA-OP6 / JB-3.1
            │   ├── AREA-OP7 / JB-3.1
            │   ├── AREA-OP8 / JB-3.1
            │   └── AREA-OP9 / JB-3.1
            ├── FRONTAL_POLE
            │   ├── AREA-FP1 / JB-3.1
            │   └── AREA-FP2 / JB-3.1
            ├── FRONTO-MARGINAL_SULCUS
            │   └── AREA-MFG2 / JB-3.1
            ├── GAPM-FRONTAL-I.1 / JB-3.1
            ├── GAPM-FRONTAL-I.2 / JB-3.1
            ├── GAPM-FRONTAL-II / JB-3.1
            ├── GAPM-FRONTAL-TO-OCCIPITAL / JB-3.1
            ├── GAPM-FRONTAL-TO-TEMPORAL-I / JB-3.1
            ├── INFERIOR_FRONTAL_GYRUS
            │   ├── AREA-44 / JB-3.1
            │   └── AREA-45 / JB-3.1
            ├── INFERIOR_FRONTAL_SULCUS
            │   ├── AREA-IFJ1 / JB-3.1
            │   ├── AREA-IFJ2 / JB-3.1
            │   ├── AREA-IFS1 / JB-3.1
            │   ├── AREA-IFS2 / JB-3.1
            │   ├── AREA-IFS3 / JB-3.1
            │   └── AREA-IFS4 / JB-3.1
            ├── LATERAL_ORBITOFRONTAL_CORTEX
            │   ├── AREA-FO4 / JB-3.1
            │   ├── AREA-FO5 / JB-3.1
            │   ├── AREA-FO6 / JB-3.1
            │   └── AREA-FO7 / JB-3.1
            ├── MEDIAL_ORBITOFRONTAL_CORTEX
            │   ├── AREA-FO1 / JB-3.1
            │   ├── AREA-FO2 / JB-3.1
            │   └── AREA-FO3 / JB-3.1
            ├── MESIAL_PRECENTRAL_GYRUS
            │   └── AREA-6MP / JB-3.1
            ├── MFG_IFS
            │   └── AREA-MFG5 / JB-3.1
            ├── MIDDLE_FRONTAL_GYRUS
            │   ├── AREA-8V1 / JB-3.1
            │   ├── AREA-8V2 / JB-3.1
            │   ├── AREA-MFG1 / JB-3.1
            │   └── AREA-MFG4 / JB-3.1
            ├── POSTERIOR_SFG
            │   └── AREA-6MA / JB-3.1
            ├── PRECENTRAL_GYRUS
            │   ├── AREA-4A / JB-3.1
            │   └── AREA-4P / JB-3.1
            ├── SUPERIOR_FRONTAL_GYRUS
            │   ├── AREA-8D1 / JB-3.1
            │   ├── AREA-8D2 / JB-3.1
            │   ├── AREA-SFG2 / JB-3.1
            │   └── AREA-SFG4 / JB-3.1
            ├── SUPERIOR_FRONTAL_SULCUS
            │   ├── AREA-6D3 / JB-3.1
            │   ├── AREA-SFG3 / JB-3.1
            │   ├── AREA-SFS1 / JB-3.1
            │   └── AREA-SFS2 / JB-3.1
            └── VENTRAL_PRECENTRAL_GYRUS
                ├── AREA-6R1 / JB-3.1
                ├── AREA-6V1 / JB-3.1
                ├── AREA-6V2 / JB-3.1
                └── AREA-6V3 / JB-3.1
```

```
BRAIN
└── TELENCEPHALON
    └── CEREBRAL_CORTEX
        └── INSULA
            ├── AGRANULAR_INSULA
            │   ├── AREA-IA1 / JB-3.1
            │   ├── AREA-IA2 / JB-3.1
            │   └── AREA-IA3 / JB-3.1
            ├── DYSGRANULAR_INSULA
            │   ├── AREA-ID1 / JB-3.1
            │   ├── AREA-ID10 / JB-3.1
            │   ├── AREA-ID2 / JB-3.1
            │   ├── AREA-ID3 / JB-3.1
            │   ├── AREA-ID4 / JB-3.1
            │   ├── AREA-ID5 / JB-3.1
            │   ├── AREA-ID6 / JB-3.1
            │   ├── AREA-ID7 / JB-3.1
            │   ├── AREA-ID8 / JB-3.1
            │   └── AREA-ID9 / JB-3.1
            └── GRANULAR_INSULA
                ├── AREA-IG1 / JB-3.1
                ├── AREA-IG2 / JB-3.1
                └── AREA-IG3 / JB-3.1
```

```
BRAIN
└── TELENCEPHALON
    └── CEREBRAL_CORTEX
        └── LIMBIC_LOBE
            ├── FRONTAL_CINGULATE_GYRUS
            │   ├── AREA-25
            │   │   ├── AREA-25.25A / JB-3.1
            │   │   └── AREA-25.25P / JB-3.1
            │   ├── AREA-33 / JB-3.1
            │   ├── AREA-P24AB
            │   │   ├── AREA-P24AB.P24A / JB-3.1
            │   │   └── AREA-P24AB.P24B / JB-3.1
            │   ├── AREA-P24C
            │   │   ├── AREA-P24C.PD24CD / JB-3.1
            │   │   ├── AREA-P24C.PD24CV / JB-3.1
            │   │   └── AREA-P24C.PV24C / JB-3.1
            │   ├── AREA-P32 / JB-3.1
            │   ├── AREA-S24
            │   │   ├── AREA-S24.S24A / JB-3.1
            │   │   └── AREA-S24.S24B / JB-3.1
            │   └── AREA-S32 / JB-3.1
            ├── HIPPOCAMPAL_FORMATION
            │   ├── AREA-EC / JB-3.1
            │   ├── HIPPOCAMPUS-CA1 / JB-3.1
            │   ├── HIPPOCAMPUS-CA2 / JB-3.1
            │   ├── HIPPOCAMPUS-CA3 / JB-3.1
            │   ├── HIPPOCAMPUS-DG / JB-3.1
            │   ├── HIPPOCAMPUS-HATA / JB-3.1
            │   ├── HIPPOCAMPUS-SUBC
            │   │   ├── HIPPOCAMPUS-SUBC.PAS / JB-3.1
            │   │   ├── HIPPOCAMPUS-SUBC.PRES / JB-3.1
            │   │   ├── HIPPOCAMPUS-SUBC.PROS / JB-3.1
            │   │   └── HIPPOCAMPUS-SUBC.SUB / JB-3.1
            │   └── HIPPOCAMPUS-TRS / JB-3.1
            ├── OLFACTORY_CORTEX
            │   ├── BASALFOREBRAIN-TU / JB-3.1
            │   └── BASALFOREBRAIN-TUTI / JB-3.1
            └── PIRIFORM_CORTEX
                ├── AREA-PIRT
                │   ├── AREA-PIRT.TIT / JB-3.1
                │   └── AREA-PIRT.TU / JB-3.1
                └── AREA-PIRTB
                    ├── AREA-PIRTB.TBD / JB-3.1
                    └── AREA-PIRTB.TBV / JB-3.1
```

```
BRAIN
└── TELENCEPHALON
    └── CEREBRAL_CORTEX
        └── OCCIPITAL_LOBE
            ├── DORSAL_OCCIPITAL_CORTEX
            │   ├── AREA-HOC3D / JB-3.1
            │   ├── AREA-HOC4D / JB-3.1
            │   └── AREA-HOC6 / JB-3.1
            ├── LATERAL_OCCIPITAL_CORTEX
            │   ├── AREA-HOC4LA / JB-3.1
            │   ├── AREA-HOC4LP / JB-3.1
            │   └── AREA-HOC5 / JB-3.1
            ├── OCCIPITAL_CORTEX
            │   ├── AREA-HOC1 / JB-3.1
            │   └── AREA-HOC2 / JB-3.1
            └── VENTRAL_OCCIPITAL_CORTEX
                ├── AREA-HOC3V / JB-3.1
                └── AREA-HOC4V / JB-3.1
```

```
BRAIN
└── TELENCEPHALON
    └── CEREBRAL_CORTEX
        └── PARIETAL_LOBE
            ├── INFERIOR_PARIETAL_LOBULE
            │   ├── AREA-PF / JB-3.1
            │   ├── AREA-PFCM / JB-3.1
            │   ├── AREA-PFM / JB-3.1
            │   ├── AREA-PFOP / JB-3.1
            │   ├── AREA-PFT / JB-3.1
            │   ├── AREA-PGA / JB-3.1
            │   └── AREA-PGP / JB-3.1
            ├── INTRAPARIETAL_SULCUS
            │   ├── AREA-HIP1 / JB-3.1
            │   ├── AREA-HIP2 / JB-3.1
            │   ├── AREA-HIP3 / JB-3.1
            │   ├── AREA-HIP4 / JB-3.1
            │   ├── AREA-HIP5 / JB-3.1
            │   ├── AREA-HIP6 / JB-3.1
            │   ├── AREA-HIP7 / JB-3.1
            │   └── AREA-HIP8 / JB-3.1
            ├── PARIETAL_OPERCULUM
            │   ├── AREA-OP1 / JB-3.1
            │   ├── AREA-OP2 / JB-3.1
            │   ├── AREA-OP3 / JB-3.1
            │   └── AREA-OP4 / JB-3.1
            ├── PARIETO-OCCIPITAL_SULCUS
            │   └── AREA-HPO1 / JB-3.1
            ├── POSTCENTRAL_GYRUS
            │   ├── AREA-1 / JB-3.1
            │   ├── AREA-2 / JB-3.1
            │   ├── AREA-3A / JB-3.1
            │   └── AREA-3B / JB-3.1
            └── SUPERIOR_PARIETAL_LOBULE
                ├── AREA-5CI / JB-3.1
                ├── AREA-5L / JB-3.1
                ├── AREA-5M / JB-3.1
                ├── AREA-7A / JB-3.1
                ├── AREA-7M / JB-3.1
                ├── AREA-7P / JB-3.1
                └── AREA-7PC / JB-3.1
```

```
BRAIN
└── TELENCEPHALON
    └── CEREBRAL_CORTEX
        └── TEMPORAL_LOBE
            ├── COLLATERAL_SULCUS
            │   └── AREA-COS1 / JB-3.1
            ├── FUSIFORM_GYRUS
            │   ├── AREA-FG1 / JB-3.1
            │   ├── AREA-FG2 / JB-3.1
            │   ├── AREA-FG3 / JB-3.1
            │   ├── AREA-FG4 / JB-3.1
            │   └── AREA-FG5 / JB-3.1
            ├── GAPM-FRONTAL-TO-TEMPORAL-II / JB-3.1
            ├── GAPM-TEMPORAL-TO-PARIETAL / JB-3.1
            ├── HESCHL_GYRUS
            │   ├── AREA-TE-1.0 / JB-3.1
            │   ├── AREA-TE-1.1 / JB-3.1
            │   └── AREA-TE-1.2 / JB-3.1
            ├── OCCIPITO-TEMPORAL_SULCUS
            │   └── AREA-OTS1 / JB-3.1
            ├── PARAHIPPOCAMPAL_GYRUS
            │   ├── AREA-PH1 / JB-3.1
            │   ├── AREA-PH2 / JB-3.1
            │   └── AREA-PH3 / JB-3.1
            ├── SUPERIOR_TEMPORAL_GYRUS
            │   ├── AREA-TE-2.1 / JB-3.1
            │   ├── AREA-TE-2.2 / JB-3.1
            │   └── AREA-TE-3 / JB-3.1
            ├── SUPERIOR_TEMPORAL_SULCUS
            │   ├── AREA-STS1 / JB-3.1
            │   └── AREA-STS2 / JB-3.1
            ├── TEMPORAL_INSULA
            │   ├── AREA-TEI / JB-3.1
            │   └── AREA-TI / JB-3.1
            └── TEMPORO-PARIETAL_JUNCTION
                └── AREA-TPJ / JB-3.1
```

```
BRAIN
└── TELENCEPHALON
    └── CEREBRAL_NUCLEI
        ├── AMYGDALA
        │   ├── AMYGDALA-CM
        │   │   ├── AMYGDALA-CM.AAA / JB-3.1
        │   │   ├── AMYGDALA-CM.CE / JB-3.1
        │   │   └── AMYGDALA-CM.ME / JB-3.1
        │   ├── AMYGDALA-IF
        │   │   ├── AMYGDALA-IF.ICE / JB-3.1
        │   │   ├── AMYGDALA-IF.IOL / JB-3.1
        │   │   └── AMYGDALA-IF.LD / JB-3.1
        │   ├── AMYGDALA-LB
        │   │   ├── AMYGDALA-LB.BL / JB-3.1
        │   │   ├── AMYGDALA-LB.BM / JB-3.1
        │   │   ├── AMYGDALA-LB.LA / JB-3.1
        │   │   └── AMYGDALA-LB.PL / JB-3.1
        │   ├── AMYGDALA-MF
        │   │   ├── AMYGDALA-MF.ICM / JB-3.1
        │   │   └── AMYGDALA-MF.LM / JB-3.1
        │   ├── AMYGDALA-SF
        │   │   ├── AMYGDALA-SF.AHI / JB-3.1
        │   │   ├── AMYGDALA-SF.APIR / JB-3.1
        │   │   └── AMYGDALA-SF.VCO / JB-3.1
        │   ├── AMYGDALA-VTM / JB-3.1
        │   └── AMYGDALOSTRIATAL_TZ
        │       └── AMYGDALA-ASTR / JB-3.1
        ├── BASAL_FOREBRAIN
        │   ├── BASALFOREBRAIN-BST / JB-3.1
        │   ├── BASALFOREBRAIN-CH-123 / JB-3.1
        │   └── SUBLENTICULAR_BASAL_FOREBRAIN
        │       └── BASALFOREBRAIN-CH-4 / JB-3.1
        └── BASAL_GANGLIA
            ├── Caudate / AAL3
            ├── N_Acc / AAL3
            ├── Pallidum / AAL3
            ├── Putamen / AAL3
            ├── VENTRAL_PALLIDUM
            │   └── VENTRALPALLIDUM-VP / JB-3.1
            └── VENTRAL_STRIATUM
                ├── VENTRALSTRIATUM-ACBL / JB-3.1
                ├── VENTRALSTRIATUM-ACBM / JB-3.1
                ├── VENTRALSTRIATUM-FUCD / JB-3.1
                └── VENTRALSTRIATUM-FUP / JB-3.1
```

References

Amunts, K., H. Mohlberg, S. Bludau and K. Zilles (2020). Julich-Brain: A 3D probabilistic atlas of the human brain's cytoarchitecture. *Science* **369**(6506): 988-992.

Benjamini, Y. and Y. Hochberg (1995). Controlling the False Discovery Rate: A Practical and Powerful Approach to Multiple Testing. *Journal of the Royal Statistical Society: Series B (Methodological)* **57**(1): 289-300.

Bogomolov, M., C. B. Peterson, Y. Benjamini and C. Sabatti (2020). Hypotheses on a tree: new error rates and testing strategies. *Biometrika* **108**(3): 575-590.

Hofhansel, L., C. Weidler, M. Votinov, B. Clemens, A. Raine and U. Habel (2020). Morphology of the criminal brain: gray matter reductions are linked to antisocial behavior in offenders. *Brain Structure and Function* **225**(7): 2017-2028.

Müller, J. L., S. Gänßbauer, M. Sommer, K. Döhnel, T. Weber, T. Schmidt-Wilcke and G. Hajak (2008). Gray matter changes in right superior temporal gyrus in criminal psychopaths. Evidence from voxel-based morphometry. *Psychiatry Research: Neuroimaging* **163**(3): 213-222.

Rolls, E. T., C.-C. Huang, C.-P. Lin, J. Feng and M. Joliot (2020). Automated anatomical labelling atlas 3. *NeuroImage* **206**: 116189.

Schneider, F., U. Habel, C. Kessler, S. Posse, W. Grodd and H.-W. Müller-Gärtner (2000). Functional Imaging of Conditioned Aversive Emotional Responses in Antisocial Personality Disorder. *Neuropsychobiology* **42**(4): 192-201.

Simes, R. J. (1986). An improved Bonferroni procedure for multiple tests of significance. *Biometrika* **73**(3): 751-754.

Tzourio-Mazoyer, N., B. Landeau, D. Papathanassiou, F. Crivello, O. Etard, N. Delcroix, B. Mazoyer and M. Joliot (2002). Automated anatomical labeling of activations in SPM using a macroscopic anatomical parcellation of the MNI MRI single-subject brain. *Neuroimage* **15**(1): 273-289.
